# Receptor Concentration and Diffusivity Control Multivalent Binding of Sv40 to Membrane Bilayers

**DOI:** 10.1371/journal.pcbi.1003310

**Published:** 2013-11-14

**Authors:** Oliwia M. Szklarczyk, Nélido González-Segredo, Philipp Kukura, Ariella Oppenheim, Daniel Choquet, Vahid Sandoghdar, Ari Helenius, Ivo F. Sbalzarini, Helge Ewers

**Affiliations:** 1 MOSAIC Group, Institute of Theoretical Computer Science and Swiss Institute of Bioinformatics, ETH Zurich, Zurich, Switzerland; 2 Laboratory of Physical Chemistry, ETH Zurich, Zurich, Switzerland; 3 Department of Haematology, Hebrew University Faculty of Medicine, Jerusalem, Israel; 4 4UMR 5297 CNRS, Universite de Bordeaux, Bordeaux, France; 5 Institute of Biochemistry, ETH Zurich, Zurich, Switzerland; Tel Aviv University, Israel

## Abstract

Incoming Simian Virus 40 particles bind to their cellular receptor, the glycolipid GM1, in the plasma membrane and thereby induce membrane deformation beneath the virion leading to endocytosis and infection. Efficient membrane deformation depends on receptor lipid structure and the organization of binding sites on the internalizing particle. To determine the role of receptor diffusion, concentration and the number of receptors required for stable binding in this interaction, we analyze the binding of SV40 to GM1 in supported membrane bilayers by computational modeling based on experimental data. We measure the diffusion rates of SV40 virions in solution by fluorescence correlation spectroscopy and of the receptor in bilayers by single molecule tracking. Quartz-crystal microbalance with dissipation (QCM-D) is used to measure binding of SV40 virus-like particles to bilayers containing the viral receptor GM1. We develop a phenomenological stochastic dynamics model calibrated against this data, and use it to investigate the early events of virus attachment to lipid membranes. Our results indicate that SV40 requires at least 4 attached receptors to achieve stable binding. We moreover find that receptor diffusion is essential for the establishment of stable binding over the physiological range of receptor concentrations and that receptor concentration controls the mode of viral motion on the target membrane. Our results provide quantitative insight into the initial events of virus-host interaction at the nanoscopic level.

**Author Summary**

Viruses cannot replicate themselves and are therefore required to enter cells and to abuse cellular resources for reproduction. The information required for viruses to enter cells is encoded in the structure of viral molecules that bind to receptors on cell surfaces. The nature of the receptor determines the route of infection of the incoming virus. A class of cancer-causing viruses and bacterial toxins bind not to protein, but to lipid receptors and share a strikingly similar, ordered array of receptor binding-sites on their surface, suggesting that they bind to several receptors. We investigated here by a combination of experimental and computational work the interaction of individual incoming SV40 virions with lipid receptors in membranes. Our results show that virions require several receptors to attach productively and that at receptor concentrations that are found in host organisms, the motion of receptors within the membrane is important for the virion to gather a sufficient number of receptors. Our computational model can be used for any type of interaction between a polyvalent ligand and mobile receptors in a surface.

## Introduction

Simian virus 40 (SV40) is a non-enveloped, DNA tumor-virus. Its capsid is assembled from 360 copies of the VP1 protein, organized into 72 pentamers in an icosahedral architecture. Incoming SV40 virions bind via VP1 to the carbohydrate moiety of the glycosphingolipid GM1 [1,2], the cellular receptor for infection [3] in the extracellular leaflet of the plasma membrane of host cells. The hydrophobic portion of GM1 controls an invagination step critical for SV40 binding-induced membrane-deformation and subsequent clathrin-independent endocytosis [4]. Binding to lipids, specifically glycolipids, is emerging as a common mechanism by which viruses and bacterial toxins exploit clathrin-independent endocytic pathways for their uptake into cells [5–9]. Since the affinity of the individual binding site is low [2], it is generally assumed that the multivalent toxins and viruses bind to several lipids, but direct experimental evidence is lacking.

The translational motion of GM1-bound SV40 particles [10] and Cholera toxin [11] in membranes is significantly slower than that of free GM1. Furthermore SV40 particles bound to GM1 in supported membrane bilayers seem to wobble in the plane of the membrane suggesting a transition from one group of receptors to another with continuous formation and breakage of bonds [12]. Multivalent binding to lipids can cause redistribution of the receptor to a different membrane phase [13] or in itself induce phase separation [14] and even membrane deformation [4,8]. However, the dynamic interaction of a polyvalent molecule in solution such as a virus with several lipid receptors dissolved in a membrane remains poorly understood because of experimental difficulties associated with its nanoscopic scale. It is unknown how many receptor lipids a virion requires for stable attachment, or whether the diffusivity of the receptor lipid, which is relatively high compared to receptor proteins of other viruses, influences viral binding.

Here we investigated the kinetics and mechanics of the interaction of SV40 with its receptor GM1 *in vitro* and *in silico*. We developed a computational model for the interaction of polyvalent virions with membrane-dissolved receptors partially based on previous work [15] with modification of several key algorithms. Specifically, we included a robust stochastic dynamics algorithm for angular rigid body motion, applied a simpler, more general virus-membrane interaction and we refined the Brownian dynamics algorithm. We calibrated our model for the SV40-GM1 system using experimental data of virus and receptor dynamics and kinetics presented here and in published work. We predicted the intrinsic rate constants for single bond formation and breakage. Our calibrated model reproduced receptor concentration-dependent attachment of SV40 virions to membranes containing diffusive receptors *in silico*.

Using this model, we found that SV40 must bind to several receptors to establish stable binding. Translational diffusion of receptors strongly influenced the binding over a range of receptor concentrations. We furthermore showed that the 3D translational and rotational motion of the membrane-bound virion changes with increasing receptor concentration. We provide here a computational model that relates the structure-based information stored in the capsid of a virus to the dynamic properties of its receptor in membrane bilayers and use it to characterize the rules of virus-receptor engagement.

## Results

### Dynamic properties of SV40 virus-like particles and GM1

**Figure 1:**
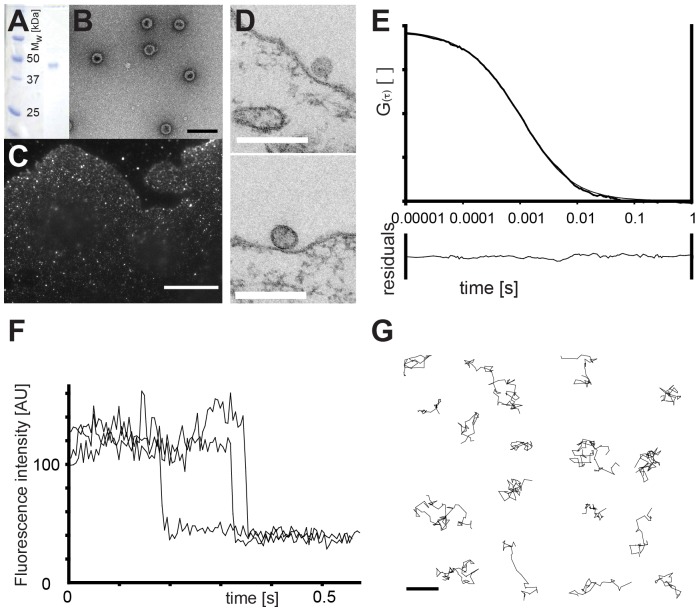
Preparation of SV40 virus-like particles and measurement of virus and receptor dynamics. (A) Recombinantly expressed, purified SV40 VP1 protein analyzed by gel-electrophoresis. Left is the marker lane, the right single band represents the VP1 protein at ∼40 kDa. (B) In vitro assembled SV40 virus-like particles (VLPs) as seen by transmission electron microscopy after negative staining. Scale bar is 100 nm. (C) Fluorescence-microscopy image of Atto-565 labeled SV40 VLPs bound to CV1 cells. Scale bar is 10 µm. (D) Electron microscopy images of SV40 VLPs immediately after binding to cellular plasma membranes. Scale bars are 200 nm. (E) Top: Fluorescence correlation spectroscopy measurement of the diffusion of Atto488-labeled SV40 VLPs in buffer solution. Shown is the overlay of the average of 5 measurements of 5 seconds each and the fit with 3D free-space diffusion model. Bottom: Residuals of the fit. (F) Fluorescence-intensity traces of individual TMR-GM1 molecules at trace amounts in a DOPC supported membrane bilayer in time-lapse acquisition showing one-step photobleaching. (G) Examples of measured single molecule trajectories of TMR-GM1. Scale bar is 1 µm.

First, we aimed to determine the dynamic properties of SV40 virions in solution and GM1 molecules in a membrane bilayer. To do so, we prepared non-infectious virus-like particles (VLPs) from recombinantly expressed capsid protein VP1 (Figure 1 A–C) [16]. These particles do not carry the SV40 genome, but retain binding properties of the virion and entered cells like intact virions [4]. When observed in electron microscopy immediately after addition to cells, they attached to flat plasma membrane (Figure 1D) before they became engulfed in tight fitting membrane invaginations in the first step of the endocytic program [4]. We next labeled the VLPs with a fluorescent dye and performed fluorescence correlation spectroscopy measurements *in vitro* to determine the diffusion coefficient of the capsid in solution. The correlation curve could be fit by a simple model for 3D-diffusion and resulted in a diffusion coefficient of 8 µm^2^/s (Figure 1E). To measure the diffusion of GM1 molecules in a membrane, we prepared supported membrane bilayers on a coverglass and incorporated into the bilayer GM1 molecules with a fluorescent dye coupled to the lipid moiety. We then mounted the bilayer on a custom-made single-molecule microscopy setup and imaged individual fluorescent molecules moving in the plane of the bilayer (Figure 1 F, G). Nearly all GM1 molecules were mobile and exhibited random motion in the membrane (not shown), the average diffusion coefficient was ∼1.8±0.8 µm^2^/s as calculated from the slope of the mean-square displacements of tens of individual traces.

### A stochastic model of virus-membrane interaction

Based on our experimental data, we developed a computational model to investigate the kinetics and mechanics of the virus-receptor attachment process. *In silico*, we could freely vary important properties of the system such as receptor density and diffusion that are difficult to control precisely *in vitro*. Furthermore, we could run thousands of individual simulations of viral attachment in order to generate statistically relevant amounts of data.

**Figure 2:**
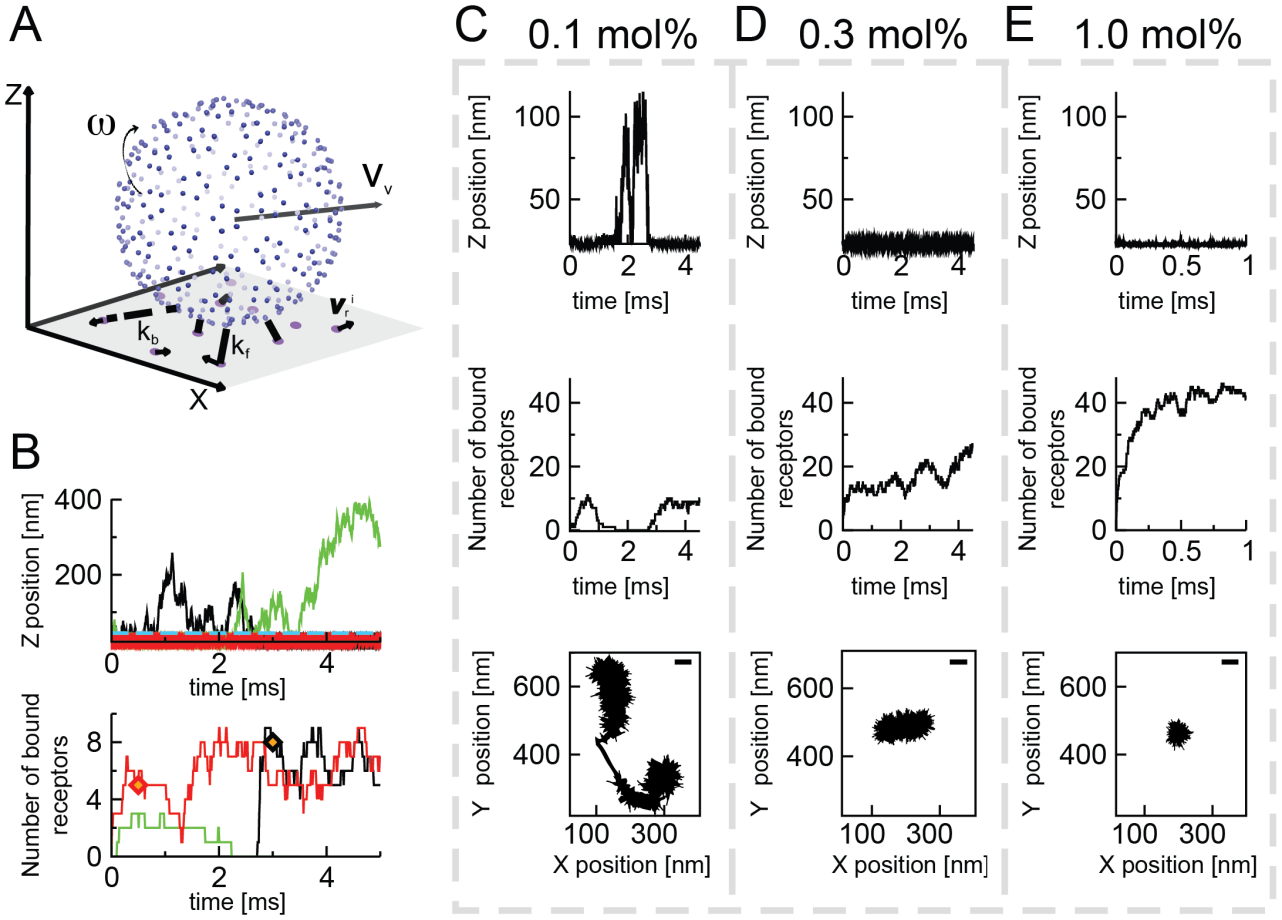
A stochastic model for virus to membrane binding. (A) A vertex representation of the viral capsid. Blue points correspond to the positions of the C-α atoms of the Ser66 residues of the VP1. The virions undergo both 3D translational (***υ*** **_v_**) and rotational motion (***ω***). The GM1 glycolipids are represented as purple points in a flat membrane (gray), undergoing 2D translational motion (***υ*** **^i^_r_**). In each time-step a bond can be formed between a ligand and a receptor (solid yellow line) with an intrinsic standard bond-forming rate *k_f_* and a previously existing bond can be broken with an intrinsic standard bond-breaking rate *k_b_* (dotted yellow line). (B) Example trajectories of the virus-membrane interaction over time for three different virions. Plotted are the distances of the virions from the membrane *z_v_(t)* (upper panel) and the number of bonds between virions and the membrane *n_br_(t)* (lower panel) over time. In the upper panel, the blue dashed line indicates the distance from the membrane equal to the virus radius (i.e., virus touching the membrane). Two of the virions (black and red lines) reach stable binding at a certain time (diamonds in the bottom graph), and one of the virions detaches from the membrane after a failed binding attempt (green line). (C–E) Characterization of the virion-membrane interaction for representative single virions over time for three different receptor concentrations: 0.1 (C), 0.3 (D), and 1.0 (E) mol% GM1. In the upper panel the distance between the center of mass of the virion and the membrane is shown, in the middle panel the evolution of the number of bonds, and in the bottom panels the corresponding *x–y* trajectories of the particles in the plane of the membrane. The scale bar is 45 nm, corresponding to the diameter of an SV40 virus particle.

Individual viral capsids were simulated in a cubic computational box with periodic boundary conditions in *x* and *y* directions. The virus capsid was generated as a structure consisting of points on a sphere, where each point represents a single GM1 binding-site in a single VP1 capsid protein. It was modeled after the X-ray structure of the SV40 VP1 protein (1SVA.pdb). The positions of the points reflect the C-α atoms of Ser66 of VP1 (Figure 2A). This serine is one of the key residues that interact with the sialic acid of the GM1 carbohydrate chain [2]. The receptors were represented as points in a flat membrane at the bottom of the simulation box (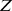 = 0 plane) and were subject to 2D translational motion. SV40 particles underwent both 3D translational and 3D rotational motion in the medium above the membrane (see *Methods*). The motion of both the virus and the glycolipid receptors was simulated by solving the stochastic dynamics equations of motion (see *Methods*). For the viral rotational motion we developed a new algorithm (*Langevin Quaternion Dynamics* – see Text S1) by combining quaternions of motion with a stochastic dynamics velocity Verlet algorithm [17]. This algorithm was crucial to ensure the conservation of the rigid body structure of the viral capsids.

Our computational model is partly based on previous work [15,18] with modifications to several key algorithms (see *Methods*). The 3D space is divided into two regions with different levels of description: the Langevin Dynamics (*LD*) regime close to the membrane and the Brownian Dynamics (*BD*) regime further from the membrane (for details, see *Methods*). In the *BD* regime the probability of forming a bond between a virus and a receptor in the membrane is lower than 10^−17^, and thus the virus particle is subject only to thermal collisions with the solvent modeled in an implicit manner, resulting in purely diffusive Brownian motion. In the *LD* regime both stochastic forces resulting from collisions with the solvent, as well as deterministic forces resulting from bonds and from collisions with the membrane act on the virion. At each time-step of the simulation when the virion is in the *LD* regime, a bond can be formed between a VP1 binding site and a GM1 receptor with an instantaneous bond-formation rate *k_f_*, and a previously existing bond can be broken with an instantaneous bond-breakage rate *k_b_*. The binding process is governed by a probability function [19] of forming or breaking a single VP1-GM1 bond (for details see *Methods*).

Among other data, the simulations generated trajectories of the 3D-position of the center of mass of the virus particle over time (***r*** *_v_*) (Figure 2B, top panel), and the corresponding time evolution of the number of receptors bound to the virus (*n_br_*) (Figure 2B, lower panel). We could observe viruses approaching the membrane, gathering a number of receptors and detaching again (green trajectory in Figure 2B) and other virions that would gather higher numbers of receptors and stay bound (red and black trajectories). When we changed the concentration of receptors in the membrane, we found that the measured parameters depend on the amount of receptor present in the target membrane (Figure 2 C–E). Specifically, we found that with increasing receptor concentration virions were in closer contact with the membrane (top panels), formed more bonds (middle panels) and became less mobile in the plane of the membrane (lower panels). We concluded that our model reproduced complex previous observations of the virus-membrane interaction, such as a receptor-concentration dependent change in the pattern of SV40 motion on membranes [10,12].

### Binding of SV40 capsids to GM1 in membranes

We validated and tested our model in comparison with *in vitro* binding assays at different concentrations. First, we performed binding assays of recombinantly expressed and purified SV40 capsids to GM1-containing lipid bilayers by quartz-crystal microbalance (QCM-D). We injected lipid mixtures based on di-oleoyl-phosphatidylcholine doped with 1, 0.2, 0.04, and 0.008 mol% of GM1 into QCM-D chambers and observed the formation of membrane bilayers (Figure S1, [20]). When identical lipid mixtures were spiked with fluorescent lipids and added to microscopy coverglass, we found that the resulting membrane bilayers were continuous and fluid as assessed by fluorescence recovery after photobleaching experiments (Figure S1). We then washed the bilayers formed in the QCM-D chambers and flowed a solution of 100 µg/ml of purified recombinant SV40 capsids past membranes of different receptor concentrations.

**Figure 3:**
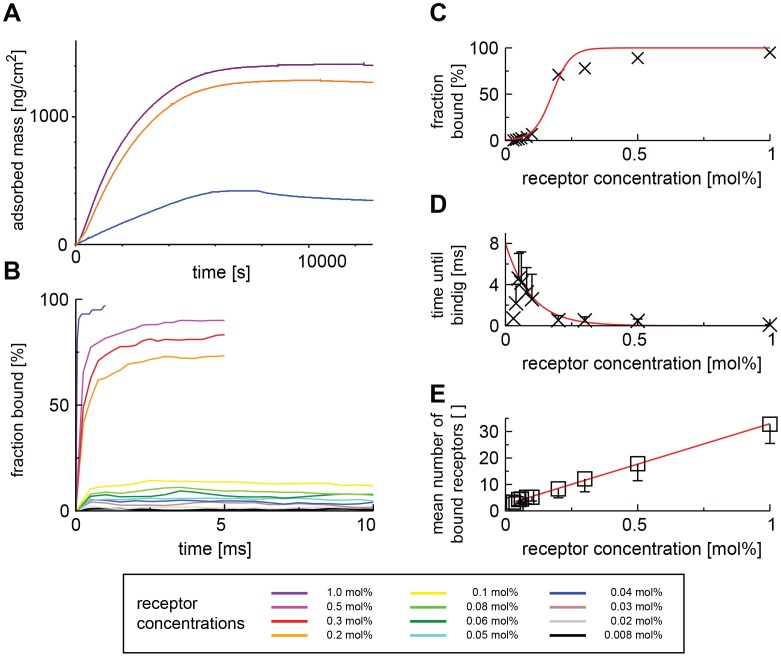
Experimental and computational characterization of the virus-receptor interaction. (A) *In vitro* binding assay of 100 µg/ml of SV40 VLPs to supported membrane bilayer containing 0.04 (blue), 0.2 (orange), and 1 mol% (purple) of GM1 in Quartz-Crystal Microbalance with Dissipation (QCM-D). (B) *In silico* binding assay. Different lines correspond to different GM1 concentrations: 0.008 (black), 0.02 (grey), 0.03 (brown), 0.04 (blue), 0.05 (light blue), 0.06 (green), 0.08 (light green), 0.1 (yellow), 0.2 (orange), 0.3 (red), 0.5 (pink), and 1.0 mol% (purple). (C–E) Results from the *in silico* binding assays: dependence of SV40 binding on receptor concentration. We show the relative amount of virions that are stably bound to the membrane (C), the mean time needed to reach that state (D), and the mean number of receptors bound to the virion in that state (E). Only data points for which stable binding was reached are included in the analysis. The inflection point from a sigmoid fit (red line, *R^2^* = 0.993166) to the fraction of stably bound viruses is at a receptor concentration of 0.17 mol%. The mean time required to reach stable binding is fit with a single-exponential decay function <*t_r_*>: *y = D(1−r)^x^* (*R^2^* = 0.623338). The first two data points were omitted for the fit, because the sampling was extremely low; the statistical meaning of those data points is thus negligible. The number of bound receptors follows a linear trend with an offset of 4–5 occupied binding sites (*R^2^* = 0.9989811).

When no receptors were present, we did not detect any binding (data not shown). In bilayers with 0.008 mol% of GM1, corresponding to 0.2 receptors per virion cross section in the plane of the bilayer, binding was just at the detectability threshold of the QCM-D. For higher concentrations we could readily observe binding (Figure 3A). We found that the amount of bound virions and the rate of attachment changed dramatically with receptor concentration. For 1 mol% and 0.2 mol% of GM1 in the bilayer, virions bound quickly at nearly identical rates and plateaued at similar levels, suggesting complete surface coverage (Figure 3A). At the same time, washing did not result in the detachment of a detectable amount of virions suggesting the formation of an extremely stable bond, likely involving multiple receptors.

When we performed the same assay on membranes containing 0.04 mol% of GM1, a concentration amounting to on average one receptor per virion cross-section in the membrane, six-fold less virions bound to the membrane than at 0.2 or 1 mol%. Taken together with the observation that washing the membrane now resulted in the detachment of a significant amount of virions, this suggested that one receptor does not suffice for stable binding. Since we did observe some stable binding, virions likely acquired additional receptors by surface diffusion after initial contact. However, since we observed only about 6-fold less binding at one receptor per viral footprint than at saturation, a fairly low number of receptors likely suffices to form a stable bond.

Our computational model allowed us to investigate the dependency of viral binding on receptor concentration in greater detail and to quantify the details of this process. As a first step, we changed the receptor concentration in our model in discrete steps (Figure 3B) and performed simulations of viral binding. To quantify the results of the thousands of individual simulations, we looked at the formation of stable bonds. A virus was considered stably bound if all of the following criteria were met under the block averaging method [21]: *i*) the virus did not detach from the membrane until the end of the simulation, *ii*) the change in the mean number of bound receptors between consecutive time-averaging blocks became smaller than the increase of bound receptors in the initial block: 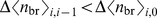, and *iii*) the virus was bound for at least 20% of the simulation time. Condition *ii*) requires the second derivative of 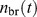 to be concave, which assures that stable attachment is defined after 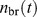 reached convergence.

We performed computer simulations for 12 different receptor concentrations (*c_r_*) between 0.008 and 1.0 mol%. The physical time window covered by the simulations depended on the receptor concentration (Table 1) in order to ensure sampling of a comparable number of binding events in all cases (see Methods). Since in the simulations the diffusion-limited step of the virion approaching the membrane is ommitted by starting the simulations with a virus particle placed close to the membrane, among other reasons, the timescales of the experiment and the simulations differ (see Figures 3A, B).

**Table 1:** Receptor concentrations (*c_r_*) simulated with the stochastic model and the corresponding simulation time windows (NT).

*c*_r_ [mol%]	0.008	0.02	0.03	0.04	0.05	0.06	0.08	0.1	0.2	0.3	0.5	1.0
NT [ms]	10	10	10	10	10	10	10	10	5	5	5	1

To generate a statistical description of the formation of stable bonds, we then analyzed our simulations according to the following parameters: *i*) the mean fraction of viruses bound, *ii*) the mean time needed to reach the state of stable attachment counted from the first time a virus-receptor bond was formed, and *iii*) the mean number of receptors bound to the virus while it is in the stably bound state.

We found that the more receptors were available in the membrane, the more virions would bind stably (Figure 3C). Remarkably, there was a clear transition between two regimes: Below 0.1 mol% of GM1 in the membrane, we observed only little binding, but already at 0.2 mol% of GM1, 75% of all virions bound stably to the membrane. A sigmoidal fit to the binding data resulted in a transition point of 0.17 mol% of GM1 in the membrane (Figure 3C, red curve). However the deviation from a sigmoidal shape indicated that the binding is not a simple one-step equilibrium but rather a multistep process. This agrees with our finding that at receptor concentrations below 0.17 mol%, virions could be washed off membranes (Figure 3A).

The time for a virion to establish a stable bond decreased rapidly with increasing receptor concentration (Figure 3D). When we analyzed the number of receptors a stably bound virion had gathered, we found that after an initial offset of 4.2, it increased linearly with receptor concentration (Figure 3E), indicating that a minimal number of receptors was required to form a stable bond.

**Figure 4:**
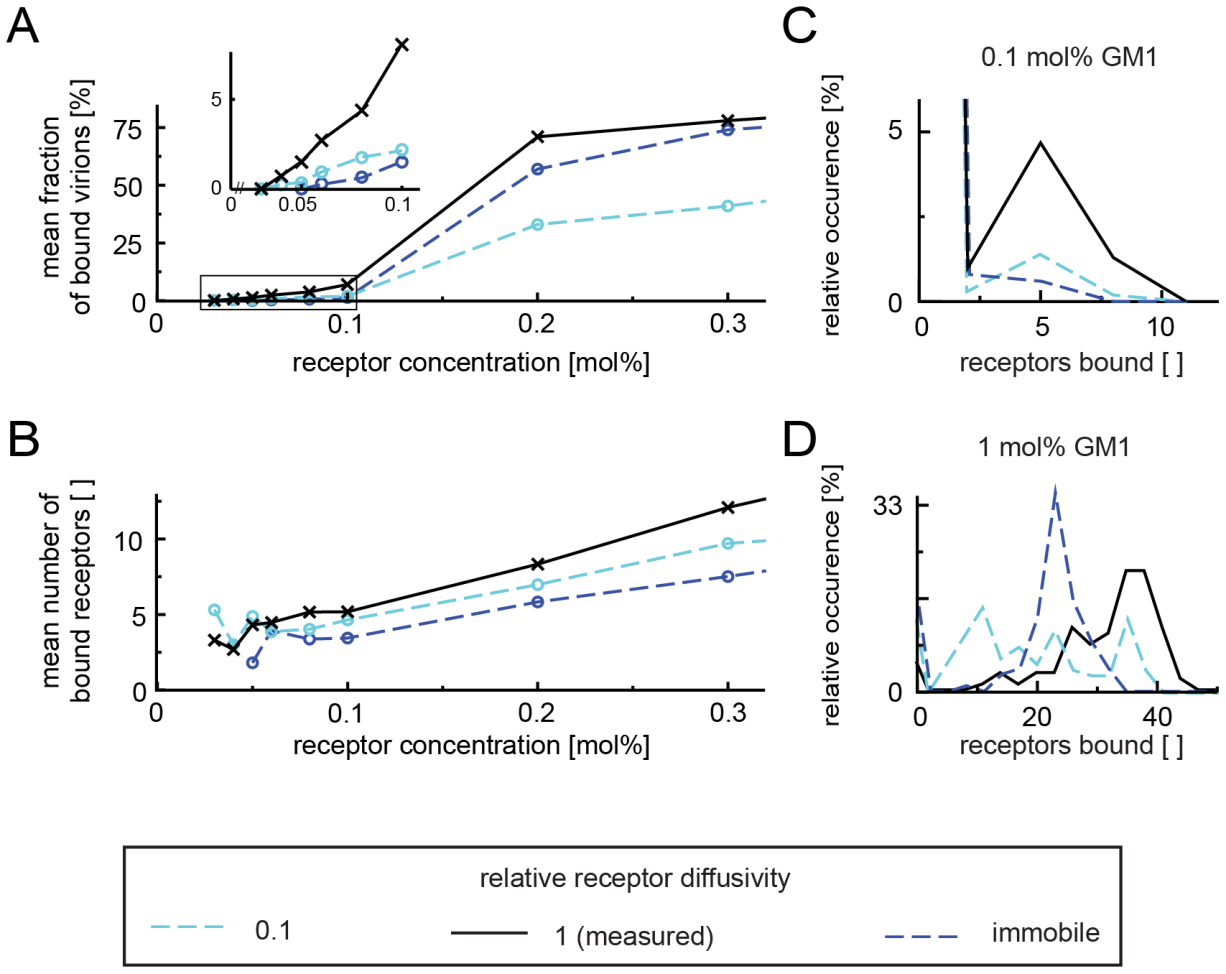
Minimum number of receptors required for stable binding and the influence of receptor diffusion on binding. The diffusivity of the receptors in the membrane was varied *in silico* from 1.8 µm^2^/s (black) to 0.18 µm^2^/s (light blue) and immobile receptors (dark blue). (**A**) Reducing the receptor diffusivity reduces the fraction of bound virions and increases the concentration at which stable binding is observed. (**B**) The mean number of stably bound receptors decreases with decreasing diffusivity of the receptors. (**C,D**) Analysis of stably bound particles for 0.1 mol% (**C**) and 1 mol% (**D**). At 0.1 mol% and the *in vitro* measured diffusivity of GM1, about 5% of particles are bound with 4–6 receptors each, but significantly less at lower diffusivities. At 1 mol%, virions gather up to 40 and more receptors for the measured diffusivity, less at 0.1 µm^2^/s and for immobile receptors a single peak is formed.

In order to analyze this finding in more detail we included simulations where the diffusivity of receptors was changed from the measured 1.8 µm^2^/s to 0.18 µm^2^/s and finally to 0. We found that the reduction in diffusion changed the fraction of bound viruses dramatically (Figure 4A), but the mean minimum number of receptors remained fairly constant at around 4 (Figure 4B). At this low concentration the influence of receptor diffusion was clearly visible. While at the measured diffusivity of 1.8 µm^2^/s, about 5% of the virions bound stably to membranes with 4–6 receptors, only about 1.8% did so for 0.18 µm^2^/s and less than 1% for immobile receptors (Figure 4C). It seemed that when receptors moved slowly, virions would unbind from the membrane before they could gather a sufficient number of receptors to form a stable bond and that the critical number was 4 receptors or more. An influence of receptor diffusivity was also obvious at higher concentrations. With immobile receptors, we observed a sharp peak at around 22 attached receptors per stably bound virion, reflecting the average density of 25 receptors per virion footprint (Figure 4D), whereas at higher diffusivity, virions could acquire more receptors (up to 39 receptors per virion). We concluded that receptor diffusion plays an important role in viral binding especially at low receptor concentrations, as it is likely to be found in cells.

**Figure 5:**
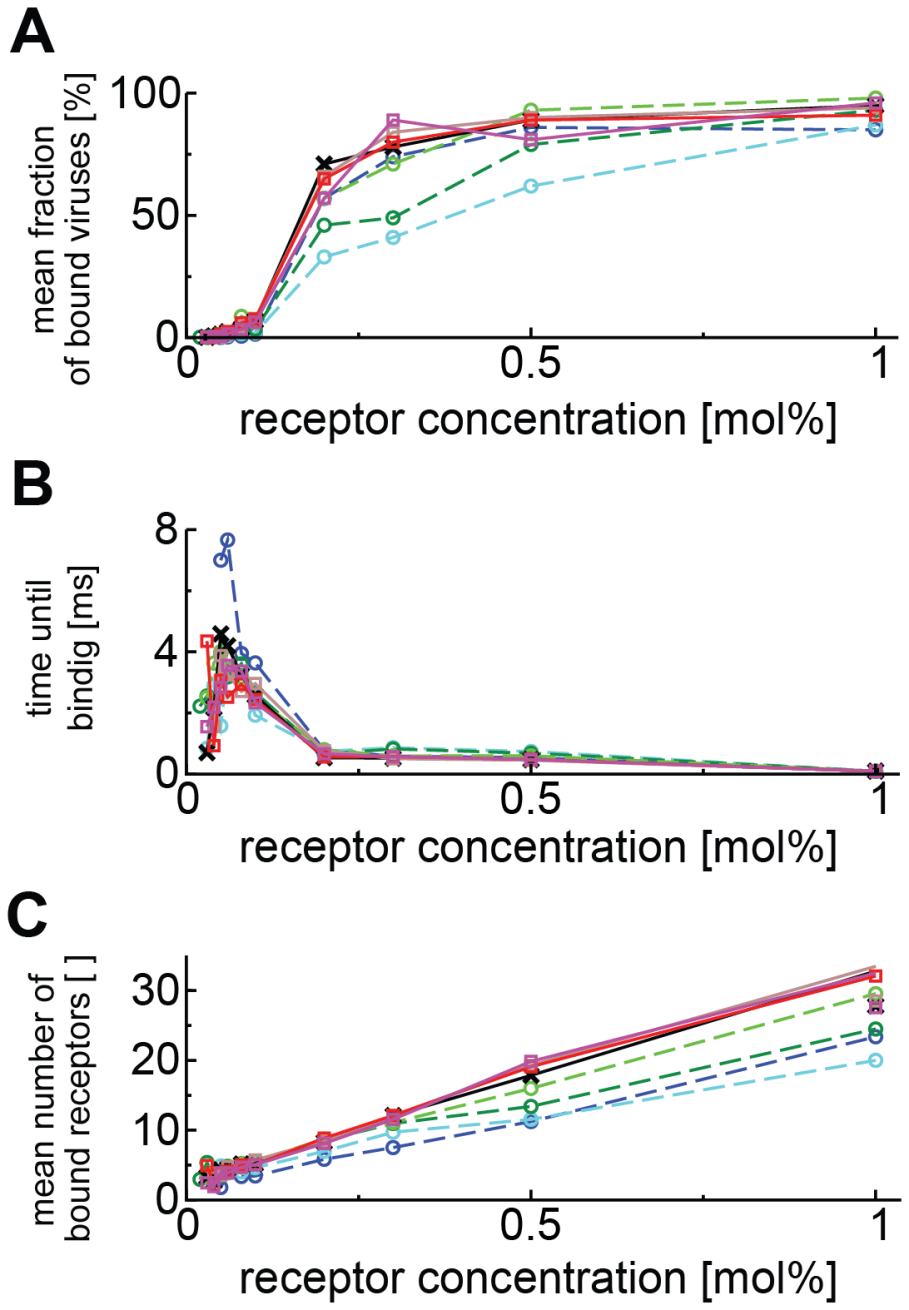
Influence of receptor diffusivity and concentration on viral binding. (**A**) Fraction of stably bound virions vs. receptor concentration for various diffusivities of GM1 in the plane of the membrane. (**B**) Average time required to reach stable binding vs. receptor concentration for various diffusivities of GM1 in the plane of the membrane. (**C**) Mean number of bonds formed by stably bound virions for various diffusivities of GM1 in the plane of the membrane. Different lines correspond to different GM1 diffusivities: 10 µm^2^/s (pink), 5 µm^2^/s (red), 2 µm^2^/s (brown), 1 µm^2^/s (black), 0.5 µm^2^/s (light green), 0.2 µm^2^/s (green), 0.1 µm^2^/s (light blue), 0 µm^2^/s (dark blue).

When we varied the diffusivity of GM1 for all receptor concentrations, we found that at the highest receptor concentration of 1.0 mol%, the bound fraction was remarkably insensitive to diffusivity (Figure 5A, see also Figure S2), as was the time until stable binding (Figure 5B). At receptor concentrations below 0.2 mol%, however, the diffusivity had a great impact on viral binding. When the receptors were immobile, only very few viruses reached stable attachment, but when receptors were mobile, virions bound stably even at low membrane concentrations (see the increasing peaks at 5 receptors per virion in Figure S2). The mean number of bound receptors was insensitive to further increasing the GM1 diffusivity above the experimental value. Importantly, receptor diffusivity did not influence the threshold of minimally 4–5 receptors required for stable binding (Figure 5C and Figure S2).

### Membrane dynamics of bound virions

In previous work [12], we found that SV40 virions that bind to membranes containing low concentrations of GM1 (0.05 mol%) undergo 3-dimensional rotation (“rolling”) and lateral motion in the plane of the membrane (“sliding”). In contrast, at high receptor concentrations (1 mol%) virions exhibited a nanoscale stepping motion, where the virion seems to rock back and forth between two adjacent pentamers. Both of these behaviors would require repeated binding and unbinding to GM1 molecules with binding sites on several pentamers. To investigate if our model can reproduce this behavior and to understand the characteristics of this type of motion, we visualized for several simulations to what extent and when exactly every individual binding site would be occupied.

**Figure 6:**
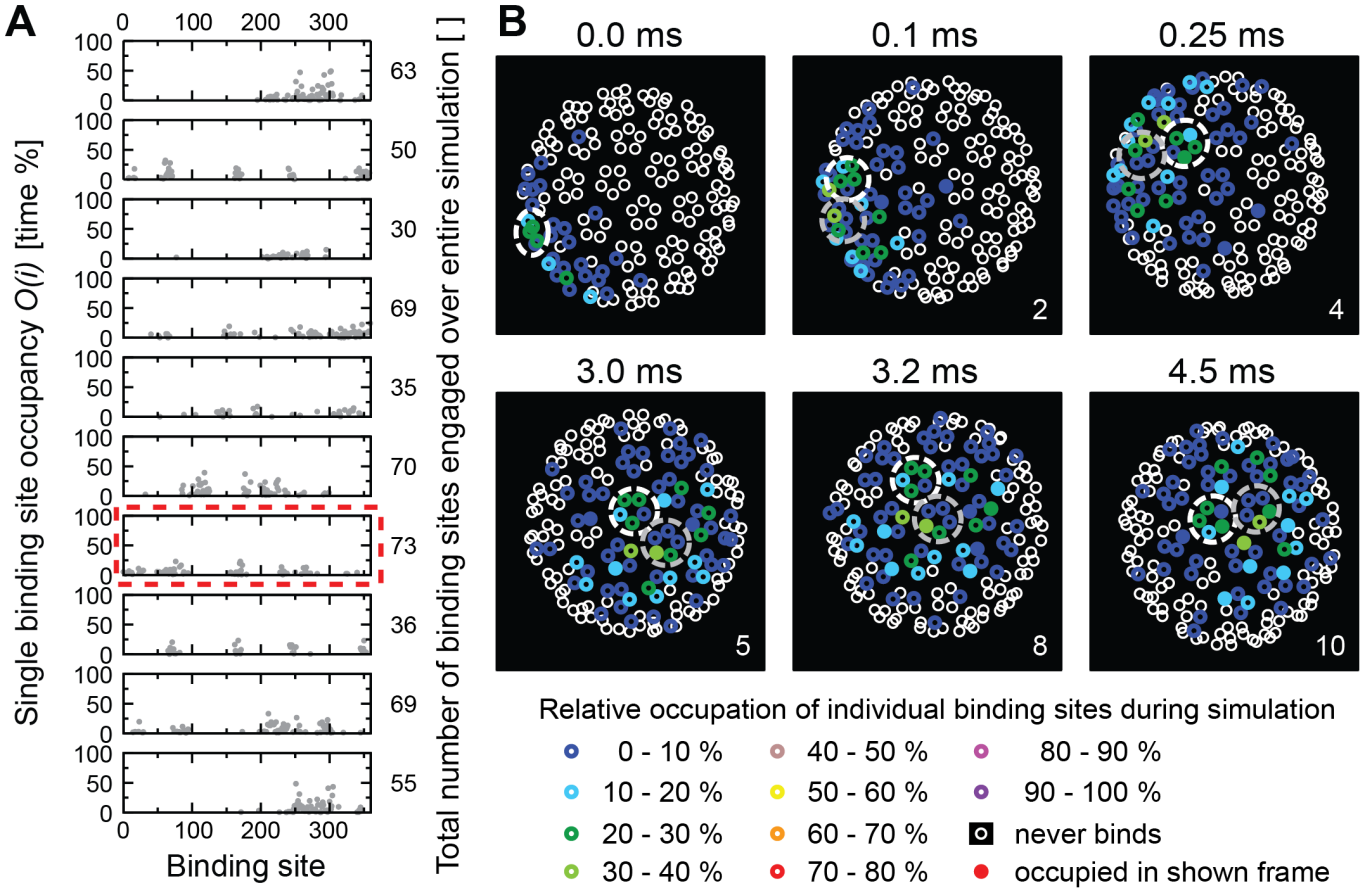
Occupancy of binding sites on a virion for 0.1 mol% GM1. (**A**) Ten graphs showing the rates at which each of the 360 individual binding sites of a virion is occupied during an entire simulation. Only binding sites with occupancy >0 are shown. The numbers on the right indicate the total numbers of binding sites that ever engaged with a receptor during the simulation. The graph emphasized by a red dashed line is analyzed in detail in (**B**). (**B**) The panels show the viral VP1 proteins projected onto the plane of the membrane as seen from below (i.e., looking up from the membrane). The white and grey dashed circles mark two adjacent pentamers to highlight viral motion. Small circles represent VP1 protein binding-sites. Sites that were never occupied by a receptor during the simulation are indicated by open white circles. Otherwise, color encodes the overall occupancy during the simulation of each VP1 protein as indicated. Sites that were bound to a receptor at the time of taking the snapshot are represented as filled circles.

We first performed such analysis for ten virions at 0.1 mol% of GM1, three of which bound stably to the membrane during the simulation, and found that while many of the 360 binding sites never interacted with a receptor, between 30 and 73 interacted with one or several different receptors over the simulation time (Figure 6A). When we then analyzed one of the ten virions in detail by visualizing the viral binding sites as viewed from the membrane, we found that over the simulation, the virion exhibited substantial 3-dimensional rotation and that a total of 28 pentamers came into contact with receptors over time (Figure 6B, see localization of white and grey dashed circles). The number of receptors increased from two to ten over the course of the simulation. To investigate this process in detail, we plotted the number of receptors bound to the virion against its localization over time (Figure S3A). The virion first came into proximity of the membrane (black circles) moved away and then at the second approach made contact to a small number of receptors (grey circles). The number of receptors bound would then fluctuate between 1 and 9 until it reached 10–15 (green circles). When we then compared the number of receptors that engaged the virion with the number of binding sites involved in this interaction, the ratio was 0.6, meaning that a small number of receptors repeatedly interacted with a larger number of binding sites. At the same time, the average interaction lasted 1.4 times longer for the receptors, meaning that the virion would drag receptors with it in the plane of the membrane. Virions were thus both sliding laterally in the membrane and rolling over the membrane by binding to new receptors, while unbinding from others.

**Figure 7:**
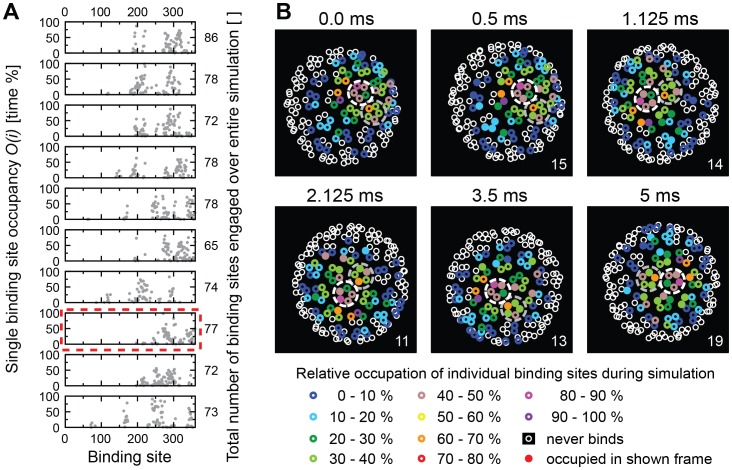
Occupancy of binding sites on a virion for 0.3 mol% GM1. (**A**) Ten graphs showing the rates at which each of the 360 individual binding sites of a virion is occupied during an entire simulation. Only binding sites with occupancy >0 are shown. The numbers on the right indicate the total numbers of binding sites that ever engaged with a receptor during the simulation. The graph emphasized by a red dashed line is analyzed in detail in (**B**). (**B**) The panels show the viral VP1 proteins projected onto the plane of the membrane as seen from below (i.e., looking up from the membrane). The white and grey dashed circles mark two adjacent pentamers to highlight viral motion. Small circles represent VP1 protein binding-sites. Sites that were never occupied by a receptor during the simulation are indicated by open white circles. Otherwise, color encodes the overall occupancy during the simulation of each VP1 protein as indicated. Sites that were bound to a receptor at the time of taking the snapshot are represented as filled circles.

When we next performed the same analysis for 0.3 mol% of receptors in the membrane (Figure 7), we found a different picture. Several binding sites were occupied for more than 50% and up to 100% of the time, and between 65 and 86 binding sites were engaged by receptors over the course of the simulation (Figure 7A). The dynamics of the virion at the membrane was different at this concentration. The overall orientation of the virion toward the membrane was more stable, exhibiting more axial spinning than lateral rolling and in the detailed analysis, only 23 pentamers overall bound to between 10 and 27 receptors (Figure 7B, Figure S3B). Immediately after entering the space close to the membrane (black circle in Figure S3B), the virion bound to a first receptor and then gathered 10 receptors within 60 µs. It slid for a considerable distance within the simulation time, but both the relative number of receptors per binding site (0.9) and the relative occupancy (1.1) of binding sites were close to unity, suggesting that the receptors traveled together with the virion through the membrane and repeatedly bound to the same group of binding sites in a stepping mode.

**Figure 8:**
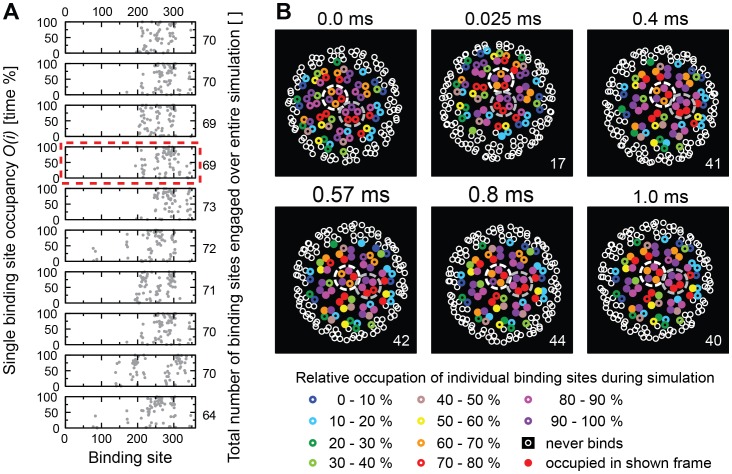
Occupancy of binding sites on a virion for 1.0 mol% GM1. (**A**) Ten graphs showing the rates at which each of the 360 individual binding sites of a virion is occupied during an entire simulation. Only binding sites with occupancy >0 are shown. The numbers on the right indicate the total numbers of binding sites that ever engaged with a receptor during the simulation. The graph emphasized by a red dashed line is analyzed in detail in (**B**). (**B**) The panels show the viral VP1 proteins projected onto the plane of the membrane as seen from below (i.e., looking up from the membrane). The white and grey dashed circles mark two adjacent pentamers to highlight viral motion. Small circles represent VP1 protein binding-sites. Sites that were never occupied by a receptor during the simulation are indicated by open white circles. Otherwise, color encodes the overall occupancy during the simulation of each VP1 protein as indicated. Sites that were bound to a receptor at the time of taking the snapshot are represented as filled circles.

When we then analyzed 10 simulations at 1 mol% GM1, we observed that several binding sites were occupied during the entire simulation and that the total number of receptors engaged during the simulation was consistently between 64 and 73 (Figure 8A). We frequently observed that entire pentamers were occupied by receptors and only 18 pentamers engaged receptors during the entire simulation, speaking for a very confined motion of the virion. This notion was supported by the binding-plot in Figure S3C, where the virion already acquired 9 receptors in the first µs, within 10 µs gathered 16 receptors, and then soon became virtually immobile with approximately 40 receptors bound. The rotational motion was reduced to a mere tumbling between pentamers, consistent with our previous experimental observations [12] (Figure 8B).

The receptor/binding-site ratio was 0.9 as for 0.3 mol% of GM1, but the occupancy ratio was only 0.6, indicating that while binding sites were mostly occupied, they frequently “side-stepped” to different (groups of) receptors. It seemed that because so many receptors were available, binding sites on pentamers were not unoccupied for sufficient durations of time to allow rolling, resulting in a tug of war between pentamers in control of the center of mass of the virion. We concluded that receptor concentration greatly influences the membrane dynamics of bound virions.

## Discussion

We investigated the binding of Simian Virus 40 to its glycolipid receptor, GM1, by a computational model. The model was based on experimental data from previous studies and data gathered in experimental work presented here. It reproduced the viral diffusion constant and the concentration-dependent attachment of SV40 virions to membranes containing diffusive receptors *in silico*. Using this model, we found that SV40 must bind to several receptors in order to establish stable binding to a membrane and that translational diffusion of receptors in the plane of the membrane strongly influences the stable binding of polyvalent particles over a range of receptor concentrations.

Our data show that after making first contact with one receptor, individual virions need to gather at least 4–5 receptors within a short amount of time to establish a stable bond. The total ganglioside content of cellular membranes can reach up to 1–2 mol%, with individual gangliosides in the range of 0.01–0.1 mol% [22–24]. This concentration range is below the transition for binding of virions, which we observe at 0.17 mol%. At physiological receptor concentrations, receptor diffusion may thus be required for stable binding. In agreement with this, at receptor mobilities significantly below measured mobilities and with immobile receptors, we detected only little binding below 0.1 mol% (Figure 4A, inset). Even tenfold reduced diffusion already led to the accumulation of receptors compared to the random distribution of immobile receptors (Figure 4C). On the other hand, as receptor concentrations increased above 0.1 mol%, slow diffusion seemed to hinder some of the viral capsids from binding. Apparently, at this concentration, the randomly generated receptor distribution at the beginning of the simulation contained clusters of sufficiently high concentration of receptors to allow some virions to bind even in the absence of diffusion. However, at a low receptor mobility, these cluster would dissipate quickly, while at the same time the slow receptor mobility would not allow a virion to gather sufficient receptors to form a stable bond. At 1 mol%, virions bound regardless of diffusion, as expected when 25 receptors per viral footprint area are present in the membrane on average. In cells, there is evidence for the local enrichment or clustering of GM1 in the plane of the plasma membrane [25,26], and SV40 may bind to such areas in cells with a globally low receptor concentration.

We furthermore found that while binding can be detected over a wide range of concentrations and diffusion rates, consistent with a wide tropism of SV40, receptor diffusion increases the amount of bound receptors and accelerates the formation of a stable bond, especially at lower receptor concentrations. We furthermore found that there is a minimal number of 4–5 receptors required to establish stable binding between the virion and the membrane, consistent with the organization of the viral binding sites into pentameric capsid subunits. This threshold is insensitive to GM1 diffusivity and concentration in the membrane, which explains the experimental observation that below certain concentrations the capsids do not bind stably.

We found that the interaction of virions with the membrane changes with increasing receptor concentration and that the relative 3-dimensional orientation of the virion on the membrane bilayer becomes more fixed with higher receptor concentrations. These findings reproduce our previous experimental observations on supported membrane bilayers [12], where we observed 3-dimensional rolling at low concentrations and a small-scale stepping motion for 1 mol% of GM1, to an astonishing extent. At the same time, they provide evidence that such a behavior may be the result of the interaction between SV40 virions and free GM1 in the membrane alone and does not require anchoring or clustering of GM1, both of which are not accounted for in our model due to the lack of receptor-receptor interactions. Here, the virions exhibit a stepping motion even when binding-sites on several pentamers are occupied.

Our model robustly produced viral binding at a range of receptor concentrations and diffusivities. However, the kinetics of reaching stable attachment was extremely sensitive to the model parameters (Figure S4). The optimal parameters for the viral attachment process must allow for the formation of elastic bonds. We found that even for low values of rate constants, when no oscillations in the number of receptors bound were observed, stable attachment did not occur if the bond was too stiff. Moreover, the bonds should be formed with sufficiently high probability, and broken only when stretched too far. The predicted intrinsic rate constant of forming a single VP1-GM1 bond was of the same order as found in previous work for the human immunodefficiency virus (HIV) [15], whereas the rate constant of bond breakage was 10^7^-fold higher. The predicted bond spring constants were 10^2^-fold lower than for HIV. Taken together, the high rate of bond breakage and the low spring constants suggest that the SV40-GM1 attachment process is very unstable and difficult to capture, especially for low receptor concentrations. This is consistent with the requirement for several receptors for the virion to form a stable bond.

Our model shows that the virion does not acquire more than about 40 receptors, consistent with the spherical shape of the virion and the lipid nature of the receptor in a flat, stiff membrane. In cells, where the membrane is more elastic, virions may gather more receptors as they wrap the membrane around themselves and internalize [4]. However, at first contact with cells, SV40 can be found attached to a flat membrane (Figure 1D), suggesting that the plasma membrane is initially stiff, and that downstream events are required to create a more flexible membrane that can be deformed by the virion.

The computational model we presented here is a general model for the interaction of polyvalent ligands with membrane receptors. It can be adapted to different receptor behaviors and different types of polyvalent ligands by modifying the initial structure and changing the parameters of the algorithms for stochastic motion and probabilistic binding. In this way, the optimal organization of binding sites on a particle could be investigated, a question that is raised by the remarkable analogy in binding-site organization between the SV40 virion and the Cholera Toxin ß subunit [9]. The present model could also be extended by introducing further parameters, such as changes in the viscosity of the medium, interaction forces between receptors (as a model for lipid domain formation), or steric interactions (collisions) between multiple viruses. Finally, this model could be used to study the SV40-GM1 interaction in even greater detail.

The main limitation of the model is that it does not include atomistic detail and thus cannot account for possible structural changes of the VP1-GM1 complex upon binding nor for the thermodynamic properties of this process, however no such structural changes have been reported [2]. Finally, the development of fluorescence correlation spectroscopy combined with stimulated emission depletion [27] and the extension of interferometric scattering (iSCAT) detection [12] may in the future allow quantitative measurement of viral binding to receptors in order to experimentally test our conclusions in live cells.

## Methods

### The computational model

Currently, there are three main approaches to model viral attachment to cell surfaces. One type of methods is based on continuous-time Markov chain models [28–30]. Those models are based on the master equation approach. Each binding site on the virus is assumed to have an equal rate of bond formation and breakage. The formation and breakage of bonds and the motion of the particles are described probabilistically. Another approach to modeling viral attachment is based on the Langevin equation of motion combined with a probabilistic binding model [15,31]. This approach accounts for the detailed motion of the virus and the membrane receptors due to deterministic forces, and for the fact that the rates of forming and breaking bonds are not equal for all viral sites. The particles feel forces resulting from the attachment (bonds are modeled as Hookean springs) and from the collisions with the solvent. For each bond that is being formed or broken, force-dependent reaction probabilities are calculated. The reaction (binding) probability depends on the distance between the reaction partners, the temperature, the intrinsic rate constants, and the bond spring and transition-bond spring constants. The intrinsic single-bond rate constants are parameters of the model and must be known from experimental data or predicted from theoretical calculations. The third approach uses coarse-grained models and molecular dynamics simulations of viral attachment and endocytosis based on purely deterministic interactions between the virion and the membrane [32].

**Figure 9:**
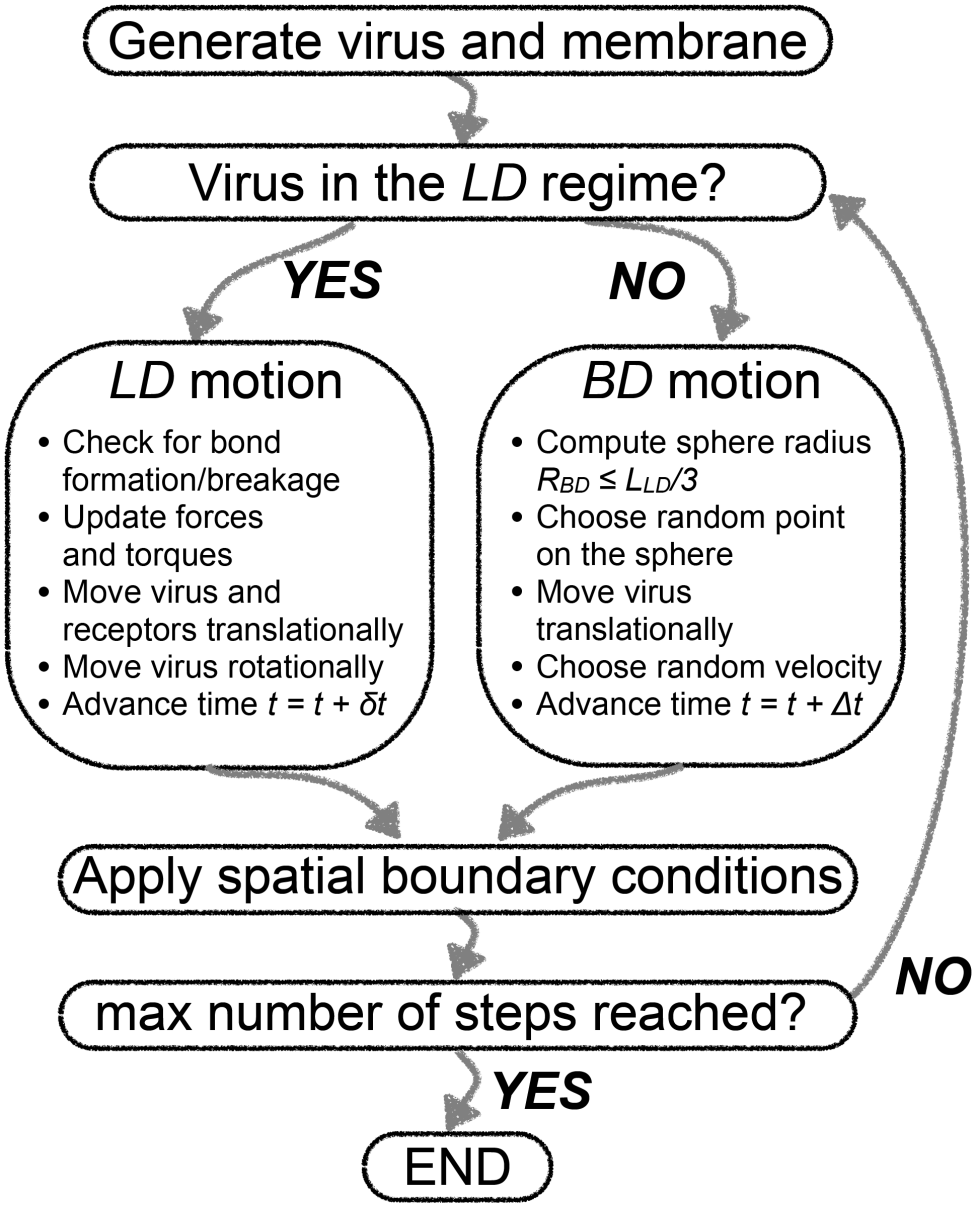
Algorithm flowchart. At each time-step, the virus' position within the computational box is determined relative to the receptor-containing membrane. If a virus particle is within the Langevin Dynamics (*LD*) region close to the membrane (below the *LD* region threshold *L_LD_*), it can stochastically bind to or unbind from one or several receptors. After computing the bond forces and torques, the translational and rotational position of the virus and the translational positions of the receptors are updated by a Langevin dynamics algorithm, and time is increased by a fixed time-step *δt*. If the virus is located in solution above a certain threshold (i.e., in the Brownian Dynamics (*BD*) regime), no binding is possible and the virus' translational position is updated in a diffusive Brownian-motion manner. Time is increased by an adaptive, distance-dependent increment *Δt*. After applying the spatial boundary conditions – periodic in *x* and *y* directions within the membrane plane and a reflection in the *z* direction orthogonal to the membrane – the cycle is repeated until the virus either leaves the computational box or time has reached a pre-defined maximum (see Table 1).

The work presented here focuses on the early events in the process of attachment of virus particles to a membrane. We thus model two regimes of motion, as in [15,18]. The 3D space was divided into two regions along the 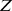 direction with a different form of the equations of motion: the Langevin Dynamics (*LD*) regime describing the details of SV40-GM1 membrane attachment close to the membrane and the Brownian Dynamics (*BD*) regime further from the membrane in which the virus particle is subject only to thermal collisions with the solvent modeled in an implicit manner resulting in purely diffusive motion (Figure 9). In the latter region, far from the receptor membrane, where the probability of forming bonds is negligible (*P_f_* <10^−17^) and the probability of breaking bonds is equal to 1, no binding can occur. When the virus particle is in the *BD* regime, a concentric sphere is constructed about the center of the particle at each simulation step. The sphere radius 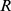
_BD_ is the shortest distance to the closest wall, either to the walls of the box or to the border of the *BD* regime. A uniformly random point is chosen on the sphere. The average time 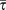 needed for the particle to diffuse to that point on the sphere is calculated from the experimentally measured diffusion coefficient *D_v_* of the particle as:
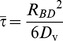
(1)This method drastically speeds up the simulation compared to a fixed time-step algorithm, since it allows the particle to move by a larger distance in one algorithm iteration. We can use this faster algorithm in the present study, because we are not interested in the details of the motion of unbound virions in solution, but rather in the kinetics of the interaction between the virion and the membrane, and in the dynamics of the viral capsids while attached to the membrane. We modified the time-adaptive algorithm with respect to the work of English and Hammer [15,18] by introducing a maximum possible sphere radius 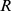
_BD,max_. The reasoning is based on the fact that for very large 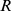
_BD_ it is possible to miss border crossings between the two regions of motion. We keep 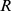
_BD,max_ equal to one third of the size of the *LD* regime in the 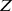 direction (see Table 2). The masses and translational diffusion constants of the virus and the receptors used in the simulations were determined in the experimental part of our work.

**Table 2:** Parameters of the stochastic computational model.

Parameter	Symbol	Value
SV40 radius^1^	*R_v_*	22.5 nm
SV40 mass^2^	*m_v_*	2.4×10^−8^ ng
SV40 diffusion coefficient^2^	*D_v_*	8×10^−3^ nm^2^/ns
GM1 mass^2^	*m_r_*	2.6×10^−12^ ng
GM1 diffusion coefficient^2^	*D_r_*	1.8×10^−3^ nm^2^/ns
Unstressed bond length^3^	*l^0^*	2.2 nm
Bond spring constant^4^	*σ*	1.2×10^−3^ N/m
Transition state bond spring constant^4^	*σ_ts_*	0.55×10^−3^ N/m
Standard intrinsic rate of bond breakage^4^	*k_b_^0^*	10^−6^ ns^−1^
Standard intrinsic rate of bond formation^4^	*k_f_^0^*	*k_b_^0^*×1.88×10^2^ ns^−1^
Simulation time-step in the *LD* regime	*δt*	1 ns
Temperature	*T*	298 K
*LD*/*BD* regime border	*L_LD_*	*R_v_+l^0^*
Cut-off radius for GM1 particles forming bonds	*R_CUT_*	4*R_v_*

1 from 1SVA.pdb.

2 determined *in vitro* in this study.

3 value determined from the crystal structure of lipid-receptor complex (PDB ID 3bwr), measured in VMD as the distance between the O1 oxygen of the BGC residue and the OG, CB and CA atoms of the Ser66 amino acid residues of the VP1 proteins.

4 determined *in silico* in this study using parameter screens as described in Supporting Tables S1 and S2.

The *LD* regime is defined by the space where the probability of forming a VP1-GM1 bond is higher than 10^−17^. In the *LD* regime both stochastic forces resulting from collisions with the solvent and deterministic forces resulting from bonds act on the particles. The bonds are modeled as springs with a resting length *l^0^* equal to the sum of the length of the VP1 portion protruding from the surface of the capsid, the length of the GM1 saccharide protruding from the membrane surface and the distance between them as calculated from the X-Ray structure (for a list of parameters, see Table 2). In order to gain detailed information about the characteristics of the attachment process, we implemented two time-stepping algorithms. The first is a modified velocity Verlet stochastic dynamics algorithm that describes the translational motion of particles [33] when no restrictions on the friction coefficient are applied [34]. The second algorithm is the *Langevin Quaternion Dynamics* algorithm used to solve the rotational motion of rigid bodies (see Text S1). We derived this algorithm by combining the modified stochastic dynamics velocity Verlet algorithm with the molecular dynamics *RVV1* algorithm for the quaternions of motion of a rigid body [17]. Several approaches for solving the rotational motion of rigid bodies using the quaternion nomenclature have been developed. Some use molecular dynamics simulations to solve the equations of motion [17] and others use hydrodynamic calculations [35]. Approaches differ also in terms of the algorithm employed [36,37]. Our modified velocity-Verlet algorithm is a simple method to solve the rotational Brownian motion of a rigid body, independent of its shape, with both deterministic and random frictional forces present in the system. No other algorithm which uses a velocity-Verlet like integration scheme for this type of stochastic dynamics motion is known to us. The derivation, testing, and properties of the rotational *LQD* algorithm itself, as well as a comparison to existing methods, are a subject of a separate publication. The steps required to solve the rotational motion of the virus particle are described in the Supplementary Text S1.

The equations of translational motion are:
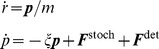
(2)
***r*** is the vector of positions, ***p*** is the vector of translational momenta, *m* is the particle mass, ***F*** ^stoch^ denotes the force vector due to the random thermal motion, and ***F*** ^det^ is the vector of deterministic forces. *ξ* is the inverse of the viscous relaxation time and is calculated using the Einstein-Smoluchowski equation [38,39]:
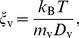
(3)where *k*_B_ is the Boltzmann constant, 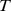 is temperature, *m*_v_ is the mass and *D_v_* is the diffusion coefficient of the virus capsid. The latter two quantities were determined in our biophysical experiments. For the receptors the inverse of the viscous relaxation time is calculated analogously:
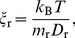
(4)where *m*_r_ and *D*_r_ are the mass and the diffusion coefficient of the receptor molecule, respectively, both determined in the experimental part of our work. The equations of motion (Eq. 2) are integrated using the modified velocity Verlet algorithm for stochastic motion with interacting particles [33,40].

The virus particles are also subject to angular motion. The rotations are modeled as motion of the ligands on the surface of the virus capsid. We use the quaternion formulation of the angular orientation of the virus capsids [41,42]:
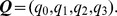
Quaternion nomenclature is derived from the Euler angles of rotation about an axis of a rigid body. Algorithms for angular motion based on quaternions are more stable than those based on Euler angles, since they do not suffer from singularities resulting from computing the sine and cosine functions. The equations of rotational motion are [33]:
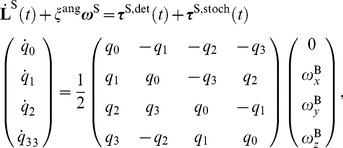
(5)where 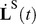 is the time derivative of the angular momentum in space-fixed coordinates and ω^S^ is the angular velocity in space fixed coordinates. *τ* ^S,det^ (t) and *τ* ^S,stoch^ (t) are the torques acting on the virus in space fixed coordinates from stochastic and deterministic forces, respectively, and are assumed to be constant during an individual simulation time-step. 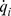 are the time derivatives of the four quaternions of motion, and 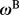 is the angular velocity of the virus in body fixed coordinates. 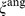 is the inverse of the viscous rotational relaxation time and is calculated from the Stokes-Einstein equation for spherical particles at small Reynolds numbers:
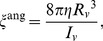
(6)where *η* is the viscosity, 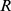
_v_ is the radius, and *I_v_* is the moment of inertia of the virus particle. The stochastic term in the total torque acting on the virus is modeled as white noise with a distribution variance defined as:
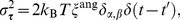
(7)where 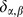 is the Kronecker delta function with *α* and *β* denoting the *x*, *y*, 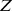 coordinates. 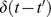 is the Dirac-delta function. We robustly solve the rotational equations of motion using a modified velocity-Verlet algorithm with quaternions (see Text S1).

The exact method used in [15] is not applicable to our virus-membrane system, since we found that solving the angular motion of the viral particles with the Euler angles method did not conserve the rigid-body structure of the capsid. The singularities in the calculation of the sine and cosine of the angular position *θ* resulted in frequent dislocation of binding sites on the capsid surface from top (*θ* = 0) to bottom (*θ* = 180), where they bound receptors and remained, effectively disrupting the structure of the capsid. As a result, the number of occupied attachment sites becomes higher than the maximum possible number as controlled by the geometry of the virion and the membrane.

#### Binding model

Binding of a virus site to a receptor is modeled as a probabilistic process [19], as in [15]. At each time-step *δt* of the simulation a bond can be formed between a VP1 binding site and a GM1 receptor with an instantaneous bond-formation constant *k_f_*, and a previously existing bond can be broken with an instantaneous bond-breakage constant *k_b_*. These instantaneous rate constants depend on system-specific parameters like the standard intrinsic rate constants of single bond formation/breakage (*k*_f_^0^, *k*_b_^0^) and the unstressed bond length (*l* ^0^). The binding process is governed by the probability distribution [19] of forming or breaking a single VP1-GM1 bond. The cumulative probabilities of forming and breaking a single bond are, respectively:
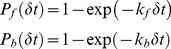
(8)The instantaneous rate constants *k*_f_ and *k*_b_ are calculated from:
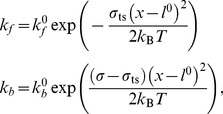
(9)where *σ* is the spring constant of the bond, *σ*_ts_ is the transition state spring constant, and *l* denotes the instantaneous bond length. For each bond formed (broken), a random number is drawn from a uniform distribution. If the number is lower than the cumulative probability of formation (breakage), an event of forming (breaking) a bond occurs and the deterministic forces are updated accordingly.

The cumulative probability of forming a new bond is computed only if a given receptor is within a certain cut-off radius *R_CUT_* around the virus and if the cosine of the angle between the ligand-to-receptor vector and the normal vector to the virus sphere at the position of the ligand is larger than 0. The first condition limits the search for bonds to only those that are spatially possible, according to the probability distribution, which significantly decreases the computational time, especially for high receptor concentrations. The second condition assures that only binding sites located on the lower half of the virus capsid with respect to the membrane can bind a receptor. Both of these conditions considerably speed up the algorithm. Moreover, each receptor and each ligand can form only one bond at a time, thus the maximum possible number of bonds that can be formed in the system is equal to half the number of viral binding sites. Bonds are modeled as springs and the resulting deterministic forces are calculated from Hooke's law and are assumed to be constant throughout a single simulation time-step:
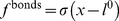
(10)


The computational model was calibrated for the SV40-GM1 system by performing a parameter screen (grid search). We varied the intrinsic rate constants (*k*_f_^0^, *k*_b_^0^) (Table S1), which to our best knowledge are not known experimentally, and the bond spring constants (*σ*, *σ*_ts_) (Table S2). The target of the model calibration for the SV40-GM1 system was to obey three conditions based on the experimental data: *i*) for receptor concentrations *c*_r_≤0.008 mol% there should be no stable binding observed, *ii*) for *c*_r_≥0.04 mol% stable binding should occur, and *iii*) for *c*_r_ = 1.0 mol% approximately all virions should be stably attached to the membrane. The final optimized values used in the simulations are summarized in Table 2. The ratio of the standard rate constants was determined from the available experimental data as [43]:
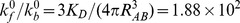
(11)where 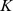
_D_ is the macromolecular equilibrium dissociation constant of a single VP1-GM1 bond and was estimated to be approximately 5 mM [2]. *R*_AB_ is the encounter distance for the ligand-receptor pair. The value of *R*_AB_ was 0.75 nm, the same as in [43] for a hapten-antibody. The dependence of the model upon changing the 4 crucial parameters listed above can be explained in terms of the cumulative probability distributions *P_f_* and *P_b_* (Equations 8 and 9). The bond spring constants *σ* and *σ*_ts_ influence the elasticity of the bond – increasing these parameters decreases the allowed strain, thus only shorter bonds can be formed (Figure S4A–C, top panels). This property of the model influences the total number of viral binding sites that can be engaged by receptors (Figure S4A–C, bottom panels), since for more elastic bonds VP1 proteins that are further from the membrane can also bind. Increasing the intrinsic rate constants of the reaction results in a higher peak in the *P_f_* distribution (Figure S4A–C, top panels, solid lines), which means that bonds form more readily. Nevertheless, the probability of breaking a bond also increases: even for the ideal bond length (no bond stretching) *P_f_* is approximately 10% (Figure S4A, top panel, dotted lines). Thus for very high rate constants bonds form with a high probability, but they also break very quickly. This model property causes strong oscillations in the time series of the number of bonds, which are especially pronounced for the highest receptor concentration (Figure S4A, bottom panels). A similar trend occurs also for lower rate constants (Figure S4B). The oscillations become smaller, but they still prevent the virus from stable attachment (Table S3). For the optimized values of *k*_f_^0^ and *k*_b_^0^ (Figure S4C) the strong oscillations disappear, the curves reach convergence, and virions bind stably to the membrane. What finally determines whether the attachment process can be reproduced by the model is a specific combination of all four parameters for which the model was screened. Only for the parameter sets presented in Table 1 the computational model met all three conditions derived from the experimental results (Figure S4C – green curves; Table S3 - results indicated in pink rectangles).

#### Spatial boundary conditions

In the *x* and *y* direction periodic boundary conditions are applied at the end of each time-step. In the 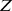 direction, the virus undergoes reflection from the box walls at 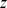 *= 0* and 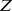 = 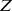
_max_. The unstressed bond length *l^0^* is a crucial parameter of the model. In our case it is very short: only 2.2 nm (Table 2) (compared to 14.5 nm in [15,18]). At the top of the box (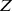 = 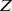
_max_), the motion of the capsids is governed by the Diffusive Brownian Dynamics algorithm, and the boundary condition is hence applied at the end of each time step. In the case of reflection from the bottom of the box (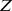 = *0*) the virus undergoes detailed stochastic dynamics as described by the Langevin Dynamics algorithm. The binding kinetics may depend on when the boundary condition is applied, since the probability of binding and the bond spring forces depend on the position of the virus with respect to the receptor (Eq's. 13, 14). The boundary conditions are hence applied right after the translational position of the virus is updated to time *t+δt*, but before the update of the angular position of the bonds.

#### Simulation details

The computational program was implemented using the *C* programming language [44] and the code is freely available under: http://www.neuro.nano-optics.ethz.ch/publications/index. At time *t* = 0 the virus particle is placed at a distance equal to the unstressed bond length *l* ^0^ from the membrane surface in order to avoid sampling the *BD* regime and to speed up the simulation. The receptors are placed randomly in the membrane at a given area concentration. For receptor concentrations up to 0.1 mol% we performed 1000 single-virus simulations with a single virus in the computational box, and for higher receptor concentrations we performed 100 single-virus simulations. The total physical time covered by a simulation varied with receptor concentration (see Table 1). This is because higher receptor densities lead to more binding and unbinding events. We thus improved the sampling of binding events for low receptor concentrations by increasing the number of simulations and the physical simulation time. Means and uncertainties of the observables were computed by ensemble and block-time averaging [21]. For the latter, trajectories were divided along time into 20 equal blocks.

## Supporting Information

Figure S1 **Bilayer formation and mobility of free GM1 in supported membrane bilayers.** (A) Fluorescence-recovery after photobleaching (FRAP) experiment of a supported membrane bilayer containing 0.1 mol% Fluorescein-di-palmitoyl-phosphatidylethanolamine and 1 mol% GM1 in di-oleoyl-phosphatidylethanolamine (DOPC). (B) Quantification of the average normalized fluorescence intensity in the photobleached spot in several experiments as shown in (A). (C) Quartz-crystal microbalance with dissipation (QCM-D) analysis of the formation of a supported membrane bilayer from vesicles containing 1 mol% GM1 in DOPC.
(TIF)

Figure S2 **Receptor diffusivity influences the distribution of the number of bound receptors.** Shown are the histograms of the number of stably bound receptors <*n_br_*> and their dependence on receptor concentration in bins of three data points. The data point <*n_br_*> = 5 on the plot thus corresponds to the sum of the occurrences of <*n_br_*> of 4–6. (A–H) Concentration-dependent binding for receptor diffusivities ranging from 0 to 10 µm^2^/s. Insets are close-ups of the data for low receptor concentrations (*c_r_*≤0.1mol%).
(TIF)

Figure S3 **Binding-dependent surface diffusion of SV40 virions.** (**A**) Lateral trajectory of the virion described in Figure 6B. The individual time-steps are color-coded according to the number of receptors bound to the virion. (**B**) Lateral trajectory of the virion described in Figure 7B. The individual time-steps are color-coded according to the number of receptors bound to the virion. (**C**) Lateral trajectory of the virion described in Figure 8B. The individual time-steps are color-coded according to the number of receptors bound to the virion. Scale bar is 100 nm.
(TIF)

Figure S4 **Properties and parametrization of the computational model.** Shown are a total of 9 out of 77 parameter sets tested. (A–C) Examples from the parametrization for different combinations of intrinsic rate constants. Top graphs: Cumulative probability distributions of forming (solid lines) and breaking (dotted lines) a single VP1-GM1 bond as a function of bond stretching. Bottom graphs: Test simulations showing the development of the number of receptors bound to a single virion over time. Different panels correspond to different sets of intrinsic rate constants [ns^−1^]: (**A**: *k*_f_^0^, *k*_b_^0^∼10^1^, 10^−1^), (**B**: *k*_f_^0^, *k*_b_^0^∼10^−2^, 10^−4^), and (**C**: *k*_f_^0^, *k*_b_^0^∼10^−4^, 10^−6^). Different colors correspond to different sets of bond spring constants [10^−3^ N/m]: (Green: *σ*, *σ*_ts_∼10^0^, 5×10^−1^), (Blue: *σ*, *σ*_ts_∼10^1^, 10^−0^) and (Red: *σ*, *σ*_ts_∼10^2^, 10^1^).
(TIF)

Table S1 **Range of standard rate constants tested to calibrate the computational model for the SV40-GM1 system.** The final values of the optimized parameters are shown in bold.
(PDF)

Table S2 **Range of bond spring constants tested to calibrate the computational model for the SV40-GM1 system.** For each pair (*k*_f_^0^, *k*_b_^0^) of rate constants (Table S1) we simulated 11 different values of bond spring constants (*σ*,*σ*_TS_). The final values of the optimized parameters are shown in bold.
(PDF)

Table S3 **Mean fraction of virions stably bound for different sets of model parameters.** Shown are the results of 9 out of a total of 77 model parameterizations for three different receptor concentrations *c*_r_ in mol%, as shown in Figure S4. The colors correspond to the values of bond spring constants as shown in the legend of Figure S4. The first row of the results corresponds to the values of rate constants as in Figure S4A. The second row of the results corresponds to the values of rate constants as in Figure S4B. The third row of the results corresponds to the values of rate constants as in Figure S4C. The results of the model with final optimized parameters are indicated in pink dashed rectangles.
(DOC)

Text S1 **The algorithm, supporting material and methods and supporting references.**
(DOC)
