# Supplementary figures and images for: Receptor Concentration and Diffusivity Control Multivalent Binding of Sv40 to Membrane Bilayers
Source: PLoS Comput Biol. 2013 Nov 14;9(11):e1003310. doi: 10.1371/journal.pcbi.1003310 (PMC3828148; doi:10.1371/journal.pcbi.1003310)

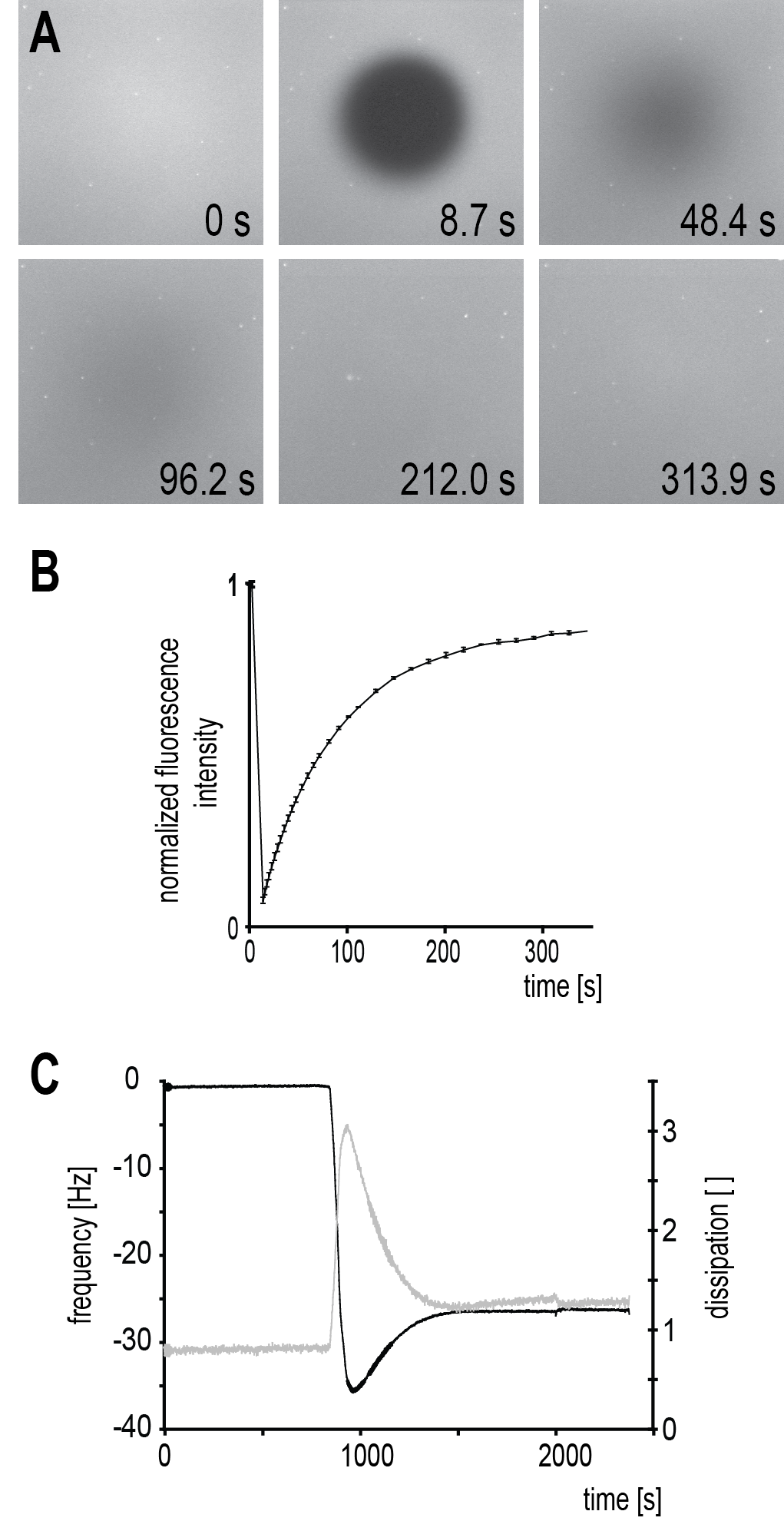

Supplement: Figure S1 — Bilayer formation and mobility of free GM1 in supported membrane bilayers. (A) Fluorescence-recovery after photobleaching (FRAP) experiment of a supported membrane bilayer containing 0.1 mol% Fluorescein-di-palmitoyl-phosphatidylethanolamine and 1 mol% GM1 in di-oleoyl-phosphatidylethanolamine (DOPC). (B) Quantification of the average normalized fluorescence intensity in the photobleached spot in several experiments as shown in (A). (C) Quartz-crystal microbalance with dissipation (QCM-D) analysis of the formation of a supported membrane bilayer from vesicles containing 1 mol% GM1 in DOPC. (TIF) [file pcbi.1003310.s001.tif]

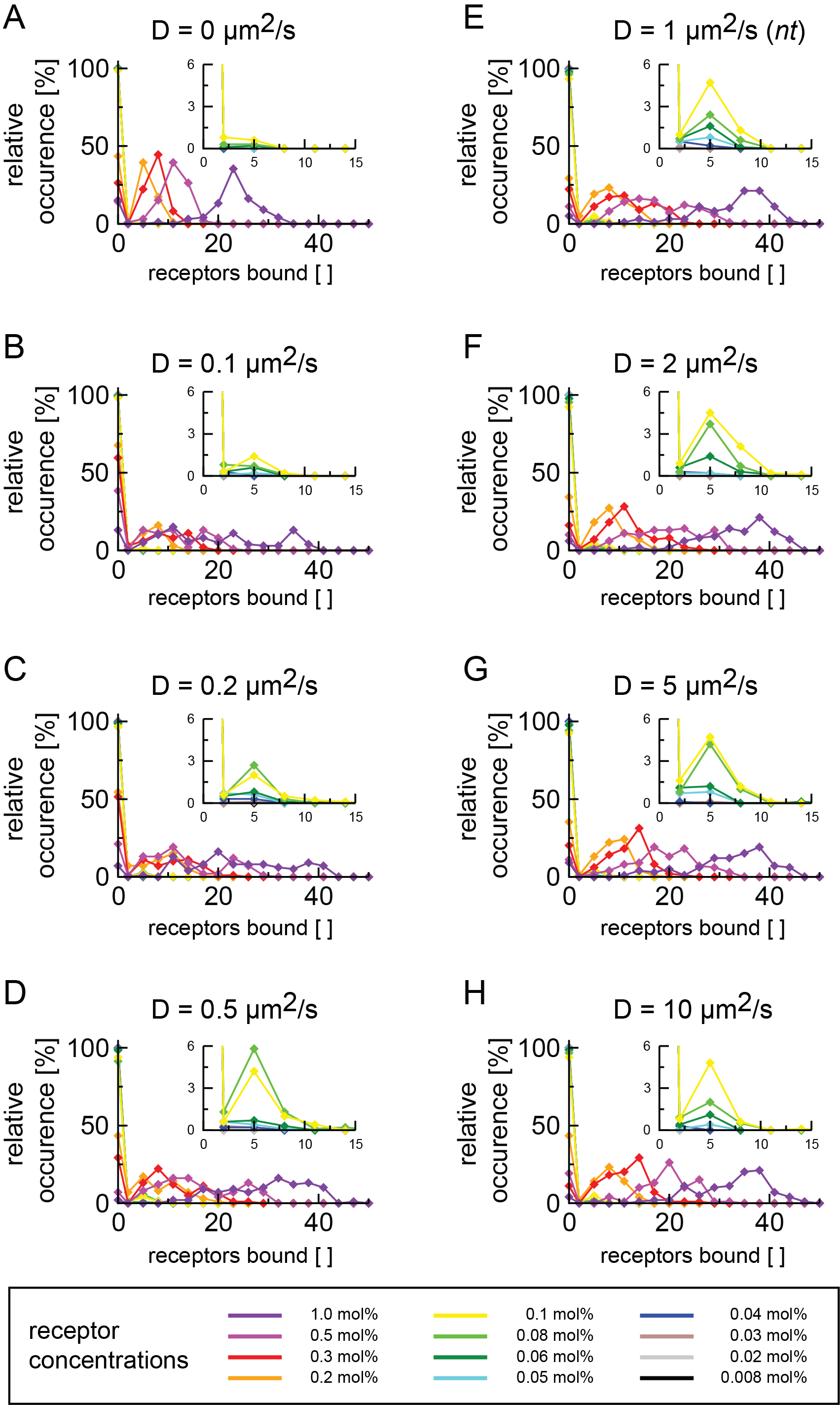

Supplement: Figure S2 — Receptor diffusivity influences the distribution of the number of bound receptors. Shown are the histograms of the number of stably bound receptors and their dependence on receptor concentration in bins of three data points. The data point = 5 on the plot thus corresponds to the sum of the occurrences of of 4–6. (A–H) Concentration-dependent binding for receptor diffusivities ranging from 0 to 10 µm2/s. Insets are close-ups of the data for low receptor concentrations (cr≤0.1mol%). (TIF) [file pcbi.1003310.s002.tif]

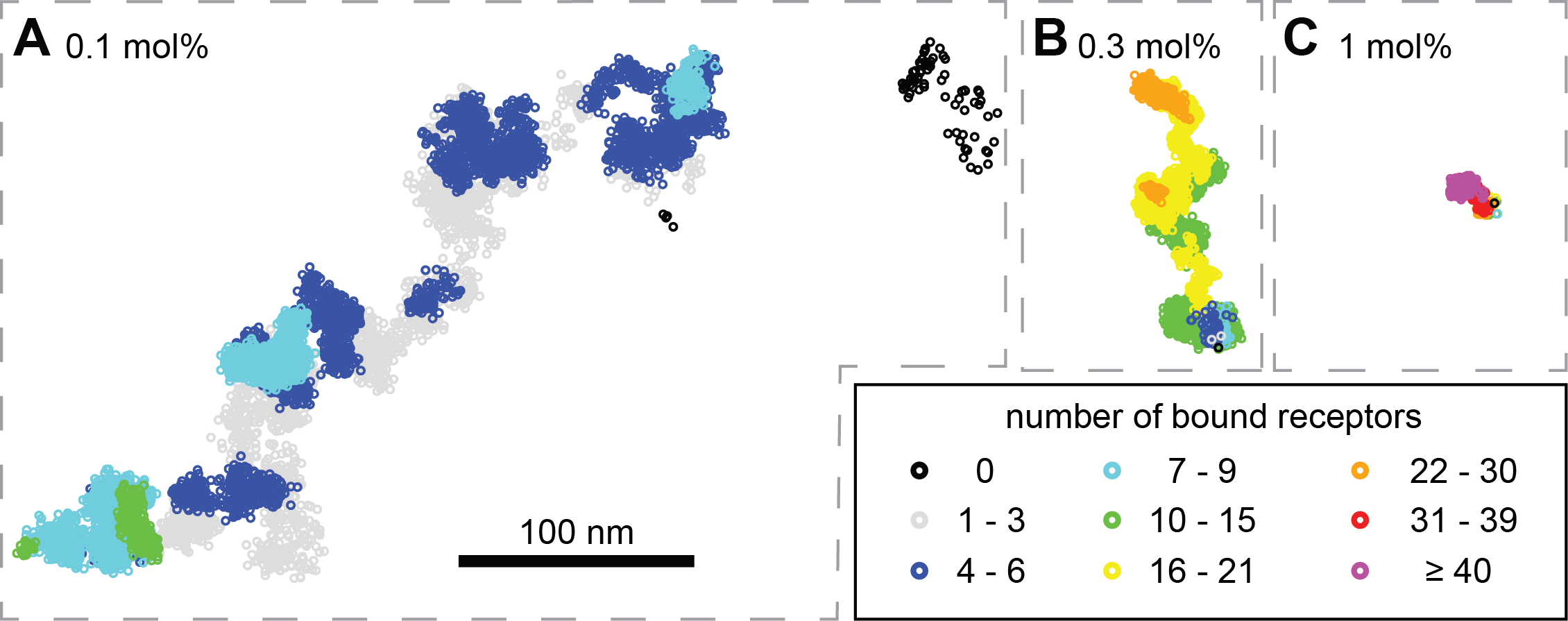

Supplement: Figure S3 — Binding-dependent surface diffusion of SV40 virions. (A) Lateral trajectory of the virion described in Figure 6B. The individual time-steps are color-coded according to the number of receptors bound to the virion. (B) Lateral trajectory of the virion described in Figure 7B. The individual time-steps are color-coded according to the number of receptors bound to the virion. (C) Lateral trajectory of the virion described in Figure 8B. The individual time-steps are color-coded according to the number of receptors bound to the virion. Scale bar is 100 nm. (TIF) [file pcbi.1003310.s003.tif]

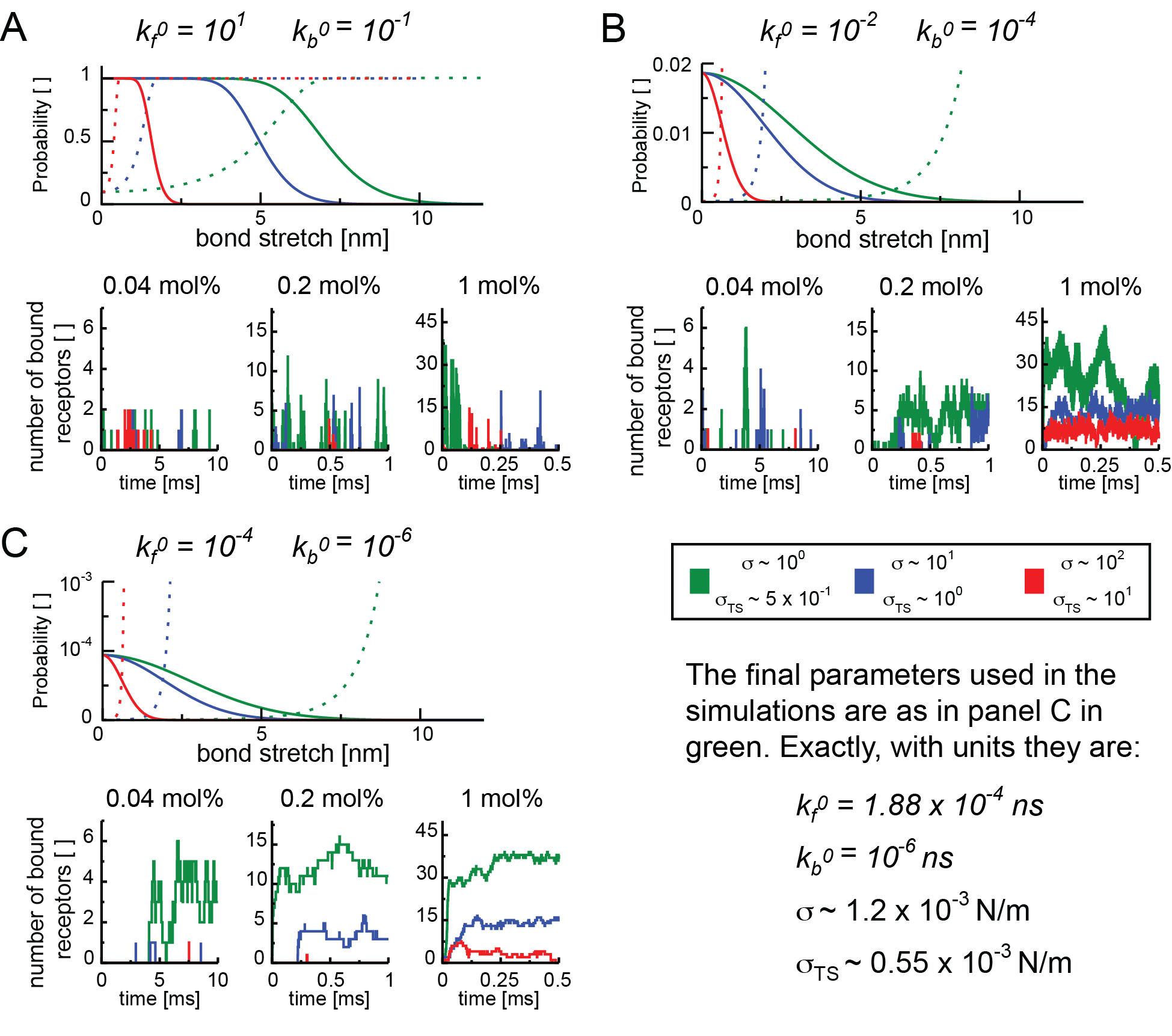

Supplement: Figure S4 — Properties and parametrization of the computational model. Shown are a total of 9 out of 77 parameter sets tested. (A–C) Examples from the parametrization for different combinations of intrinsic rate constants. Top graphs: Cumulative probability distributions of forming (solid lines) and breaking (dotted lines) a single VP1-GM1 bond as a function of bond stretching. Bottom graphs: Test simulations showing the development of the number of receptors bound to a single virion over time. Different panels correspond to different sets of intrinsic rate constants [ns−1]: (A: k f 0, k b 0∼101, 10−1), (B: k f 0, k b 0∼10−2, 10−4), and (C: k f 0, k b 0∼10−4, 10−6). Different colors correspond to different sets of bond spring constants [10−3 N/m]: (Green: σ, σ ts∼100, 5×10^−1^), (Blue: σ, σ ts∼101, 10−0) and (Red: σ, σ ts∼102, 101). (TIF) [file pcbi.1003310.s004.tif]
