# Supplementary material for: Receptor Concentration and Diffusivity Control Multivalent Binding of Sv40 to Membrane Bilayers
Source: PLoS Comput Biol. 2013 Nov 14;9(11):e1003310. doi: 10.1371/journal.pcbi.1003310 (PMC3828148; doi:10.1371/journal.pcbi.1003310)
Supplement: Table S1 — Range of standard rate constants tested to calibrate the computational model for the SV40-GM1 system. The final values of the optimized parameters are shown in bold. (PDF) [file pcbi.1003310.s005.pdf]

Table S1: Range of standard rate constants tested to calibrate the computational model for the SV40-GM1 system. The final values of the optimized parameters are shown in bold.

k_f^0 [1/1.88ns^{-1}]	10^{-5}	$\mathbf{10^{-4}}$	10^{-3}	10^{-2}	10^{-1}	10^{0}	10^{1}
k_b^0 [ns^{-1}]	10^{-7}	$\mathbf{10^{-6}}$	10^{-5}	10^{-4}	10^{-3}	10^{-2}	10^{-1}
