# Supplementary material for: Receptor Concentration and Diffusivity Control Multivalent Binding of Sv40 to Membrane Bilayers
Source: PLoS Comput Biol. 2013 Nov 14;9(11):e1003310. doi: 10.1371/journal.pcbi.1003310 (PMC3828148; doi:10.1371/journal.pcbi.1003310)
Supplement: Table S2 — Range of bond spring constants tested to calibrate the computational model for the SV40-GM1 system. For each pair (k f 0, k b 0) of rate constants (Table S1) we simulated 11 different values of bond spring constants (σ,σ TS). The final values of the optimized parameters are shown in bold. (PDF) [file pcbi.1003310.s006.pdf]

Table S2: Range of bond spring constants tested to calibrate the computational model for the SV40-GM1 system. For each pair (k_f^0, k_b^0) of rate constants (Table S1) we simulated 11 different values of bond spring constants (σ,σ_{TS}). The final values of the optimized parameters are shown in bold.

σ [1.2×10^{-3} N/m]	10^{-2}	10^{-2}	10^{-1}	10^{-1}	10^{0}	**10^{0}**	10^{0}	10^{1}	10^{1}	10^{2}	10^{1}
σ_{TS} [1.1×10^{-3} N/m]	10^{-3}	10^{-2}	10^{-2}	10^{-1}	10^{-1}	**0.5**	10^{0}	10^{0}	10^{1}	10^{1}	10^{2}
