# Supplementary material for: Receptor Concentration and Diffusivity Control Multivalent Binding of Sv40 to Membrane Bilayers
Source: PLoS Comput Biol. 2013 Nov 14;9(11):e1003310. doi: 10.1371/journal.pcbi.1003310 (PMC3828148; doi:10.1371/journal.pcbi.1003310)
Supplement: Table S3 — Mean fraction of virions stably bound for different sets of model parameters. Shown are the results of 9 out of a total of 77 model parameterizations for three different receptor concentrations c r in mol%, as shown in Figure S4. The colors correspond to the values of bond spring constants as shown in the legend of Figure S4. The first row of the results corresponds to the values of rate constants as in Figure S4A. The second row of the results corresponds to the values of rate constants as in Figure S4B. The third row of the results corresponds to the values of rate constants as in Figure S4C. The results of the model with final optimized parameters are indicated in pink dashed rectangles. (DOC) [file pcbi.1003310.s007.doc]

**Table S3. Mean fraction of virions stably bound for different sets of model parameters.** Shown are the results of 9 out of a total of 77 model parameterizations for three different receptor concentrations *c*_r_ in mol%, as shown in Figure S4. The colors correspond to the values of bond spring constants as shown in the legend of Figure S4. The first row of the results corresponds to the values of rate constants as in Figure S4A. The second row of the results corresponds to the values of rate constants as in Figure S4B. The third row of the results corresponds to the values of rate constants as in Figure S4C. The results of the model with final optimized parameters are indicated in pink dashed rectangles.

|  |  | **Stably bound virions [%] at:** | | | | | | | | |
| --- | --- | --- | --- | --- | --- | --- | --- | --- | --- | --- |
|  | ***c*_r_** | **0.04 mol%** | | | **0.2 mol%** | | | **1.0 mol%** | | |
| *σ, σ*_ts_ Graph in:  *k*_f_^0^*, k*_b_^0^ | | 10^0^, 5×10^-1^ | 10^1^, 10^-2^ | 10^2^, 10^1^ | 10^0^, 5×10^-1^ | 10^1^, 10^-2^ | 10^2^, 10^1^ | 10^0^, 5×10^-1^ | 10^1^, 10^-2^ | 10^2^, 10^1^ |
| Fig S4A | 10^1^, 10^-1^ | 0 | 0 | 0 | 0 | 0 | 0 | 0 | 0 | 0 |
| Fig S4B | 10^-2^, 10^-4^ | 0 | 0 | 0 | 20 | 0 | 0 | 90 | 70 | 20 |
| Fig S4C | 10^-4^, 10^-6^ | 10 | 0 | 0 | 70 | 20 | 0 | 100 | 70 | 30 |
