# Supplementary material for: Receptor Concentration and Diffusivity Control Multivalent Binding of Sv40 to Membrane Bilayers
Source: PLoS Comput Biol. 2013 Nov 14;9(11):e1003310. doi: 10.1371/journal.pcbi.1003310 (PMC3828148; doi:10.1371/journal.pcbi.1003310)
Supplement: Text S1 — The algorithm, supporting material and methods and supporting references. (DOC) [file pcbi.1003310.s008.doc]

**Text S1:**

**Rotational Algorithm Steps**

The *Langevin Quaternion Dynamics* algorithm we derived to treat the rotational motion of the virus particle is described here. The steps of the modified velocity-Verlet algorithm together with the equations, parameters and set-up described in the *Methods* section of the main text are sufficient to reproduce the results of our simulations. The following integration scheme of the rotational equations of motion was used:

1. Obtain the rotation matrix **RM***(t)* for quaternions as described in [1].
2. Progress angular momentum to time *t+δt/2*:

with:

$$\begin{matrix} C_{0}^{\text{ang}}=\exp\left(-\xi^{\text{ang}}\delta t\right) \\ C_{1}^{\text{ang}}=\left(1-C_{0}^{\text{ang}}\right)/\left(\xi^{\text{ang}}\delta t\right) \\ C_{2}^{\text{ang}}=\left(1-C_{1}^{\text{ang}}\right)/\left(\xi^{\text{ang}}\delta t\right) \end{matrix}$$

1. Compute angular velocity ***ω***_i_^B^ of the virus particle in body fixed coordinates at *t+δt/2*.
2. Obtain the (0)-th approximate quaternion time derivatives and (0)-th approximate quaternions of the virus particle at time *t+δt/2*:

1. Solve self-consistently the system of constraining equations for $\mathop{Q}\limits^{\bullet} \left(t+\frac{\delta t}{2}\right)$:

until the condition $\left|{}^{{}^{(k+1)}}Q\left(t+\frac{\delta t}{2}\right)-{}^{{}^{(k)}}Q\left(t+\frac{\delta t}{2}\right)\right|$ < *ε* is fulfilled, where *ε* is a convergence criterion.

1. Obtain the quaternions of motion at time *t+δt* using the final value of $\mathop{Q}\limits^{\bullet} \left(t+\frac{\delta t}{2}\right)$from step (V) of the algorithm:

$$Q\left(t+\delta t\right)=Q\left(t\right)+\delta t\mathop{Q}\limits^{\bullet} \left(t+\frac{\delta t}{2}\right)$$

1. Obtain the updated rotation matrix **RM***(t+δt)*.
2. Obtain updated positions of the viral particles VP1 in space and body fixed coordinates at time *t+δt* by performing rotation operations.
3. After updating the forces and torques acting from the bonds, progress the angular momentum in space fixed coordinates to time *t+δt* and repeat the cycle for the next simulation step:

**Supporting Material and Methods**

**Preparation of SV40 VLPs**

SV40 VLPs were generated as described previously [2] from a lysate of SF9 cells that expressed SV40 VP1. The lysate contains fully assembled SV40 VLPs as well as assembly intermediates and unassembled VP1 protein. To purify the fully assembled VLPs, the lysate was clarified by centrifugation for 30 min at 10’000 x g in an Eppendorf microfuge. The clarified supernatant (0.5 ml) was centrifuged at 4 °C for 2.5 h at 160’000 x g using a SW41Ti rotor (Beckman) through a 5 - 20 % (w/v) linear sucrose gradient with a 0.5 ml 60 % (w/w) sucrose cushion in 10 mM HEPES, pH 8.0, 200 mM NaCl, 1 mM CaCl_2_. After fractionation, 0.5 ml fractions were analyzed by transmission electron microscopy following negative staining, and fractions with homogenous intact particle populations were pooled. The purified particles were dialyzed against virus buffer and stored at – 80 °C.

**Labeling of SV40 VLPs.**

Alexa Fluor 488 was covalently coupled to SV40 VLPs in 200 mM NaHCO_3_, pH 8.2 using a 5-fold molar excess of the dye relative to VP1 protein, and resulting in less than 200 fluorophore molecules per VLP as determined according to the manufacturer’s instructions. Unbound dye was removed by chromatography with a Nap-5 column (GE Healthcare).

**Preparation of Supported membrane bilayers**

Supported membrane bilayers were formed from di-oleoyl-phosphatidycholine (Avantilipids, USA) by the vesicle drop method. Different concentrations of GM1 (Hytest, Finnland) were incorporated as indicated and for FRAP experiments trace amounts of fluorescein-di-palmitoyl-phosphatidylethanolamine were included. The Lipid mix was dried to a film under a stream of clean, dry nitrogen and kept under vacuum over night to remove traces of solvent. After hydration for at least 1 h in bilayer buffer (200 mM NaCl, 2 mM CaCl_2_, 50 mM HEPES pH 6.8) at 1 mg/ml the solution was sonicated for 30 s at full power with a probe sonicator. The vesicle solution was brought to 50 µg/ml and added to coverglass that was cleaned with 2 % Hellmanex and air plasma for microscopy experiments or injected onto SiO_2_ coated QCM-D chips cleaned with 2 % SDS over night and UV-ozone for 1 h.

**Single molecule Tracking of GM1**

Supported membrane bilayers were doped with tetramethyl-rhodamine-GM1 (TMR-GM1) by adding highly diluted TMR-GM1 to freshly prepared supported membrane bilayers. After 30 minutes of incubation, excess TMR-GM1 was washed away by several changes of bilayer buffer. Single TMR-GM1 molecules were imaged on a custom-built single molecule setup by excitation via a 543 nm laser and projection onto an Andor iXon EMCCD camera. Single molecules were detected and tracked using the software described before [3,4], freely available from *mosaic.mpi-cbg.de*.

**Fluorescence Correlation Spectroscopy of SV40 VLPs**

Fluorescence correlation spectroscopy of SV40 VLPs was performed in solution (200 mM NaCl, 2 mM NaCl, 50 mM HEPES, pH 6.8, 0.002 % Tween20) on an inverted Leica TCS SP2 confocal microscope with a Leica FCS module. The beam was focused through a Leica 100x 1.4 NA oil-immersion objective 5 µm above the coverglass and the signal of diffusing AlexaFluor 488 labeled SV40 VLPs was measured for 5 s with a 5 µs time bin. Five measurements were averaged and fit to a single exponential 3D free diffusion model.

**QCM-D experiments**

A Q-sense E-4 quartz microbalance with dissipation (QCM-D) instrument (Q-Sense, Sweden) was used to measure in situ the changes in mass (measured by the frequency shift Δ*f* ) and in viscoelasticity (measured by the dissipation factor *D*) achieved through the formation of supported membrane bilayers on the surface of an oscillating crystal and subsequent binding of SV40 VLPs. Prior to mounting in the liquid-exchange cell of the instrument, the silica (silicon dioxide)-coated AT-cut quartz crystals were cleaned by immersion in 2 % w/w SDS for a minimum of 30 min, then rinsed in Milli-Q water and dried under a stream of nitrogen. This was followed by a UV/ozone treatment (30 min). Resonance frequencies were measured at several harmonic overtones (with overtone numbers of 3, 5, and 7). The surfaces were prepared in temperature stabilized degassed bilayer buffer, and vesicle solution was added at 50 µg/ml in bilayer buffer for bilayer formation. The formation of supported membrane bilayers was monitored in the frequency and dissipation channels. Right after membrane formation was complete, excess vesicles were rinsed away by injection of 1 ml of bilayer buffer. The crystals were then exposed to constant tangential flow at a rate of 0.05 ml/min.

**Supporting References**

1. Allen MP, Tildesley DJ (1987) Computer simulation of liquids. Oxford [England] : Clarendon Press ; New York : Oxford University Press.

2. Roitman-Shemer V, Stokrova J, Forstova J, Oppenheim A (2007) Assemblages of simian virus 40 capsid proteins and viral DNA visualized by electron microscopy. Biochem Biophys Res Commun 353: 424–430. doi:10.1016/j.bbrc.2006.12.038.

3. Sbalzarini IF, Koumoutsakos P (2005) Feature point tracking and trajectory analysis for video imaging in cell biology. J Struct Biol 151: 182–195. doi:10.1016/j.jsb.2005.06.002.

4. Ewers H, Smith AE, Sbalzarini IF, Lilie H, Koumoutsakos P, et al. (2005) Single-particle tracking of murine polyoma virus-like particles on live cells and artificial membranes. Proc Natl Acad Sci USA 102: 15110–15115. doi:10.1073/pnas.0504407102.
